# Myocardial hypothermia increases autophagic flux, mitochondrial mass and myocardial function after ischemia-reperfusion injury

**DOI:** 10.1038/s41598-019-46452-w

**Published:** 2019-07-10

**Authors:** Stefanie Marek-Iannucci, Amandine Thomas, Jean Hou, Annunziata Crupi, Jon Sin, David J. Taylor, Lawrence S. Czer, Fardad Esmailian, Robert M. Mentzer, Allen M. Andres, Roberta A. Gottlieb

**Affiliations:** 1 Smidt Heart Institute, Cedars-Sinai Medical Center, Los Angeles, California USA; 2 Department of Pathology, Cedars-Sinai Medical Center, Los Angeles, California USA; 3 Division of Cardiac Surgery, Cedars-Sinai Medical Center, Los Angeles, California USA; 4 Board of Governors Regenerative Medicine Institute, Cedars-Sinai Medical Center, Los Angeles, California USA

**Keywords:** Cardiac regeneration, Heart failure

## Abstract

Animal studies have demonstrated beneficial effects of therapeutic hypothermia on myocardial function, yet exact mechanisms remain unclear. Impaired autophagy leads to heart failure and mitophagy is important for mitigating ischemia/reperfusion injury. This study aims to investigate whether the beneficial effects of therapeutic hypothermia are due to preserved autophagy and mitophagy. Under general anesthesia, the left anterior descending coronary artery of 19 female farm pigs was occluded for 90 minutes with consecutive reperfusion. 30 minutes after reperfusion, we performed pericardial irrigation with warm or cold saline for 60 minutes. Myocardial tissue analysis was performed one and four weeks after infarction. Therapeutic hypothermia induced a significant increase in autophagic flux, mitophagy, mitochondrial mass and function in the myocardium after infarction. Cell stress, apoptosis, inflammation as well as fibrosis were reduced, with significant preservation of systolic and diastolic function four weeks post infarction. We found similar biochemical changes in human samples undergoing open chest surgery under hypothermic conditions when compared to the warm. These results suggest that autophagic flux and mitophagy are important mechanisms implicated in cardiomyocyte recovery after myocardial infarction under hypothermic conditions. New therapeutic strategies targeting these pathways directly could lead to improvements in prevention of heart failure.

## Introduction

To improve neurologic outcome and recovery after cardiac arrest and successful resuscitation, therapeutic hypothermia (TH) has been an established treatment for decades^1^. TH has been shown to reduce myocardial infarction (MI) size, no-reflow and remodeling, while increasing myocardial healing and function in various animal models^2–5^. Multiple techniques, such as topical TH, regional cooling, coronary cold perfusion or whole-body temperature reduction in animals, have shown similar results with the common endpoint that moderate TH (32–34 °C) is beneficial for myocardial recovery post MI^5^. Unfortunately, clinical trials failed to achieve significant reduction in MI size, although they reported a trend to reduction of heart failure (HF) development^6,7^. TH has been demonstrated to reduce no-reflow and increase microvascular perfusion, both known as important predictors of outcome after MI^8,9^. The feasibility of TH implementation in the clinical cardiology setting remains uncertain; nonetheless, TH represents an important tool to investigate the molecular mechanisms responsible for its beneficial effect, and for discovery of potential new therapeutic strategies targeting a reduction of adverse remodeling post MI. The latter is associated with poor prognosis, progressive HF, recurrent hospitalizations and mortality^10^. Autophagy and mitophagy are important pathways implicated in remodeling and their dysfunction has been shown to enhance HF^11–16^. A pro-autophagic state seems beneficial in the failing heart, and well-functioning mitochondria are undoubtedly necessary for myocardial function^11–13^. Mitophagy is responsible for the elimination of damaged mitochondria, a mechanism necessary for intact myocardial function^11^. Its upregulation in damaged cardiac tissue has been shown to increase cellular function by reducing reactive oxygen species (ROS) and apoptosis, while increasing the functionality of mitochondria^12^. The homeostasis of well-functioning mitochondria is crucial to a healthy myocardium; therefore, equilibrated mitophagy is suspected to play a major role in prevention of remodeling and HF^14,15^. Additionally, dysfunctional autophagy itself has been related to inducing HF under ischemic conditions^13^. Notably, autophagic flux reduction has been shown to exacerbate cardiac remodeling and therefore promote cardiomyocyte deterioration^13,16,17^. In this study, we applied local moderate TH to the heart of female farm pigs through pericardial irrigation. The aim of this study was to investigate whether TH induced mitophagy and autophagy, which might explain its beneficial effects on remodeling post MI.

## Methods

### Animal model

**Figure 1:**
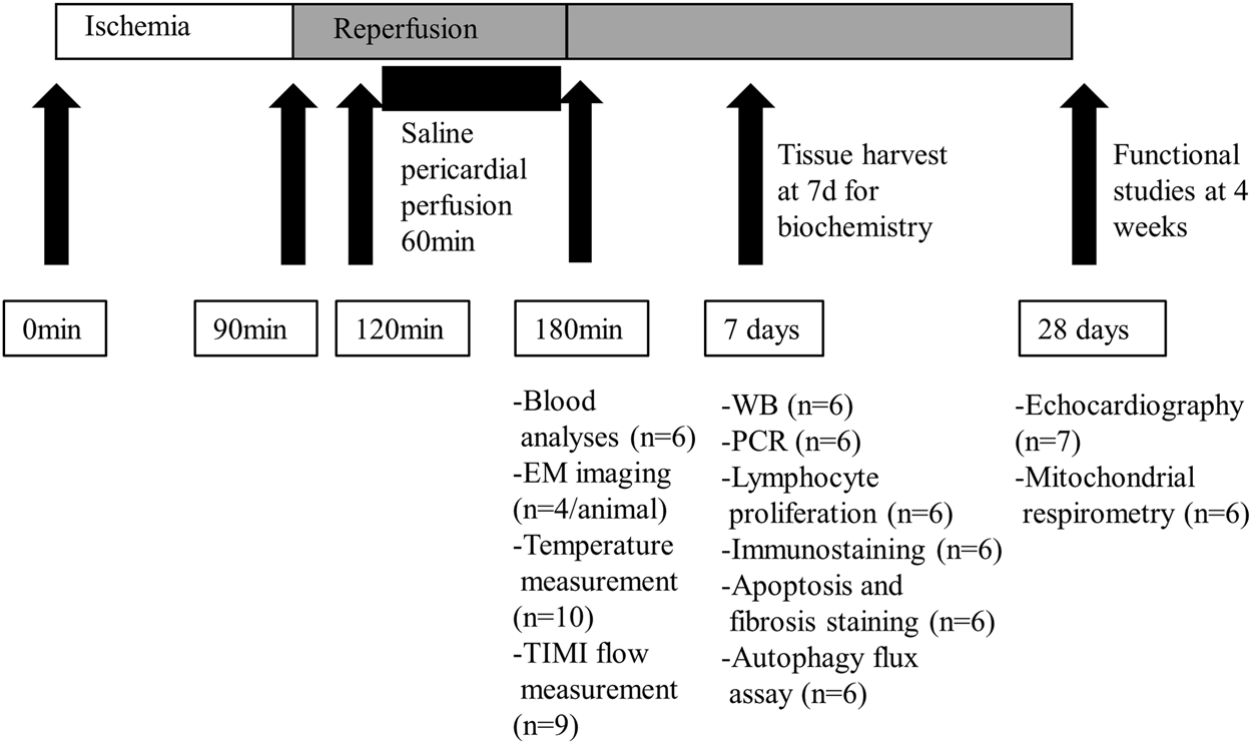
Graphical representation of the performed procedures and analyses. Figure 1 graphically represents the time course of the performed procedures. Furthermore, it indicates the different tissue harvests with the corresponding analyses performed at that time point.

All procedures and protocols were approved by the Institutional Animal Care and Use Committee (Protocol number IACUC007541) and were performed in accordance with the National Institutes of Health (NIH) Guidelines for the Care and Use of Laboratory Animals. To clarify our study protocol, Fig. 1 represents the time line of each experiment performed during this study. A total of nineteen female farm pigs (35–40 kg) were initially included into the study and randomly assigned to a warm or cold group. The major cause of death was arrhythmia, with no deaths occurring during the follow-up period. In all procedures we encountered arrhythmia within 15–45 minutes after MI, treated with intravenous pharmacological treatment or defibrillation. Unfortunately, in 6 animals the arrhythmia could not be terminated, and the animals died within the first 45 minutes of MI. Therefore, our survival rate was 70%, resulting in a final number of thirteen animals completing the study protocol. Since the affected animals died prior to treatment, and we performed one animal per day, there is no difference in the two groups. Anesthesia was induced with Ketamine (20 mg/kg IM), Acepromazine (0.25 mg/kg IM), Atropine (0.05 mg/kg IM) and Propofol (2.0 mg/kg IM) and further maintained with Isoflurane 1–3%. Vital parameters were continuously monitored (Vmed Technology, Mill Creek, WA, USA). Under anesthesia, we occluded the left anterior descending (LAD) coronary artery below the first diagonal branch with a 3.0 mm coronary balloon inflated to 2–4 atm. Complete occlusion was documented via dye injection. After 90 minutes of ischemia, the balloon was deflated under fluoroscopic control and the LAD reperfused. Within the next 30 minutes, we performed a percutaneous sub-xiphoidal pericardial puncture and inserted a 12Fr dual lumen dialysis catheter (Mahurkar-Elite, Medtronic®, Dublin, Ireland) under fluoroscopic control. Finally, the pericardium was irrigated for 60 minutes with sterile saline (37 °C in the warm and 8–11°C in the cold group) with a velocity of 1250 ml/h. Systemic temperature was measured with a rectal probe, and local myocardial temperature with a thermocouple probe (ThermoWorks, American Fork, UT, USA), inserted percutaneously into the anterolateral left ventricular wall under fluoroscopic control. After completion of the procedure (total of 3 h) the animals were taken to recovery. Blood was collected 3 h post MI and prior to euthanizing the animals for biochemical analyses one week (n = 9) and myocardial function 4 weeks (n = 10) after MI. Ischemia/reperfusion injury led to formation of three distinct areas in the myocardium (normal, border and scar).

Tissue analysis demonstrated that TH influenced only the border area (injured but still viable tissue), whereas the normal (no ischemic injury) and the scar (dead tissue) were not affected. Therefore, all the data of this study was produced using myocardium from the border area of the left ventricle (LV). The distinction of the 3 regions was rigorously made during tissue dissection, in identical manner for each animal (Supplemental Fig. 1).

### Electron microscopy (EM)

To obtain fresh tissue for EM imaging we performed a myocardial biopsy of the apical third of the interventricular septum of the left ventricle. We therefore used a myocardial biopsy forceps, which was introduced through the arterial access of the carotid artery 3 hours after infarction under fluoroscopic control. The portions of fresh tissue were immediately fixed in 3% glutaraldehyde, embedded in plastic, and cut into ultrathin sections. Sections were then stained with uranyl acetate and lead citrate and examined in a JEOL JEM-1010 or 100CX transmission electron microscope (JEOL Ltd., Tokyo, Japan). Four randomly chosen sections for each animal were analyzed by a blinded pathologist.

### Echocardiography

Echocardiography was performed with a GE Vivid 7 Ultrasound machine (Boston, MA, USA). All measurements were repeated 3 times and the mean ± standard error of the mean (SEM) was used for analysis. Ejection fraction (EF) was determined with the Simpson (2D, volume measurement) and Teichholz method (M-Mode, diameter measurement). Fractional shortening (FS) was measured with M-Mode. For diastolic function E’, E/A, E/E’ and left atrial pressure (LAP) were measured with pulse wave doppler (PW) and tissue velocity index (TVI)^18^.

### RNA isolation and mRNA quantification

RNA was isolated from tissue using miRNeasy micro Kit (QIAGEN, Hilden, Germany). We added 10 µg of Luciferase  plasmid cDNA to each sample before isolation. We used iScript™ RT-qPCR and SYBR® Green real-time PCR kit (BIO-RAD, Hercules, CA, USA). For quantification, mRNA expression of tissue necrosis factor alpha (TNFα) (5′CCTGCTGCACTTTGGAGTGA3′, 5′GAGGGTTTGCTACAACATGGG3′), transforming growth factor beta (TGFβ) (5′TGAGGGCTTTCGCCTTAGC3′, 5′GGTAGTGAACCCGTTGATGT3′), mitochondrial transcription factor A (TFAM) (5′TGCTTTGTCTACGGGTGCAA3′, 5′ACTTCCACAAACCGCACAGA3′), nuclear respiratory factor (NRF) 1 (5′GAAGCTGTCCAGGGGCTTTA3′, 5′ATCCATGCTCTGCTACTGGG3′) and NRF 2 (5′GACTCAAGGGGTTGCGAAGG3′, 5′CCCAAACCCCAATCGCGTAG3′) were normalized to Luciferase (5′AGGTCTTCCCGA CGATGA3′, 5′GTCTTTCCGTGCTCCAAAAC3′).

### Lymphocyte proliferation

Lymphocytes were isolated from splenic tissue and stimulated with gradually increasing concentrations of Concanavalin-A (Sigma-Aldrich, St. Louis, MI, USA). Proliferation was determined 48 h later with CellTiter 96® Aqueous non-radioactive cell proliferation assay (Promega, Madison, WI, USA).

### Blood analysis

Blood was collected 3 h after MI for troponin I, creatine kinase and lactate dehydrogenase analysis through the Pathology & Laboratory Medicine department of Cedars-Sinai Medical Center (CSMC).

### Western blot

We used a previously published protocol to prepare whole lysate and mitochondrial fractions of fresh and frozen tissue for western blot (WB) analysis^19^. All mitochondrial extractions from pig tissue were performed immediately after tissue harvest with fresh tissue. For our human data we isolated mitochondria from frozen tissue. The crude mitochondrial fraction of all samples was isolated with the same protocol. The tissue was cut into small pieces and immersed in HES buffer and homogenized using a Polytron. We then performed a 1000 g centrifugation for 5 minutes at 4 °C to remove tissue debris and nuclei. A fraction of the supernatant was retained for analysis of whole lysate; the remainder was centrifuged at 7000 g for 5 minutes at 4 °C. The supernatant was stored for further analysis of the crude cytosolic fraction. The remaining pellet was cautiously washed with HES buffer and centrifuged again with 7000 g for 5 minutes at 4 °C. Finally, the supernatant was discarded and the pellet resuspended in RIPA buffer for further analysis of the crude mitochondrial fraction.

The membranes were incubated with antibodies against microtubule-associated proteins 1A/1B light chain 3B (LC3) (Cell Signaling Technology #4108 S), S6 (Cell Signaling Technology #2217), phospho-S6 (Ser 235/236) (Cell Signaling Technology #2211), glucose-regulated protein 78 (GRP78) (Santa Cruz Biotechnology, #sc-376768), nuclear domain 10 protein (NDP52) (Proteintech #12229-1-AP), optineurin (Santa Cruz Biotechnology #sc-166576), p62/SQSTM1 (Abcam #ab56416), oxphos cocktail (Abcam #ab110413), autophagy related gene (APG) 5/12 complex (Santa Cruz Biotechnology #sc-133158), heat shock protein (HSP) 70 (Santa Cruz Biotechnology #sc-32239), HSP 90 (Santa Cruz Biotechnology #sc-13119), parkin (Santa Cruz Biotechnology #sc-32282), translocase of outer mitochondrial membrane (TOM) 70 (Santa Cruz Biotechnology #390545), arginase-1 (BD Bioscience #BD610708), inducible nitric oxide synthase (iNOS) (BD Bioscience #BD610332), phospho-ubiquitin (Ser65) (EMD Millipore #ABS1513-I), phosphorylated nuclear factor ‘kappa-light-chain-enhancer’ of activated B-cells (pNFkB) (Invitrogen #MA5-16160), caspase-1 (Invitrogen #PA5-20113). All proteins were normalized to ponceau (Sigma-Aldrich, St. Louis, MI, USA). We normalized each protein to the entire corresponding lane on the ponceau-stained membrane, representing the total protein amount. Due to limitation of space we show cropped gels/blots and representative ponceau in the figures. Full-length gels/blots are included in supplementary data.

### Immunohistochemical staining

Paraffin-embedded sections of the border area were deparaffinized and rehydrated by putting the slide in (1) xylene 2 times for 10 minutes each, (2) 100% ethanol 2 times for 10 minutes each, (3) 95% ethanol for 5 minutes, (4) 70% ethanol for 5 minutes. Slides were then put in deionized H_2_O for 10 minutes. A PAP pen was used to create a hydrophobic border around the sections. Slides were then placed in a 1x citrate buffer pH 6.0 (Sigma-Aldrich, Carlsbad, CA, USA) and processed for heat-induced antigen retrieval. Samples were then left to cool down at room temperature for 20 minutes. After washing with water, a blocking step was carried out using blocking buffer (5% horse serum (SH30074.03, GE Healthcare Biosciences Corp) + 0.1% Triton-X100 (Sigma-Aldrich, Carlsbad, CA, USA) + 1% BSA (Sigma-Aldrich, Carlsbad, CA, USA) in PBS (Mediatec Inc, Manassas, VA, USA)), for 1 hour at RT in a humidified chamber. The paraffin-embedded sections were then treated with anti-Arginase-1 (1:100, BD Biosciences, BD610708) and anti-iNOS (1:100, Novus Biologicals, NB300-605). Secondary staining was performed using anti-rabbit-Alexa Fluor 647 (Thermo Fisher Scientific, A32733) and anti-mouse-Alexa Fluor 594 (Thermo Fisher Scientific, A-11032). Sections were counterstained in DAPI (BIO-RAD, 1351303). Tissues were coverslipped using fluorescent mounting medium (DAKO, GM30411-2). Fibrosis and apoptosis were quantified using Picrosirius Red Stain Kit (Polysciences, Warrington, PA, USA)^20^ and ApoTag (EMD Millipore, Billerica, MA, USA) respectively, following manufacturer’s instruction.

### Image quantification

We analyzed seven pig hearts in total (3 from the warm and 4 from the cold group). For each animal we used 3 sections containing two samples each. 10x images of random unique fields per sample were acquired. For Arginase-1& iNOS 9 fields, within the inflamed area, whereas for apoptosis 5 fields within the inflamed area were examined. The inflamed area was identified within the DAPI channel as the area enriched in nuclei. On the other hand, for fibrosis 6 fields within the entire section area were examined. Images were acquired using a Leica DM6000 B fluorescent microscope, through LAS X v.2 (Leica Microsystems Inc., Buffalo Grove, IL, USA). Same parameters were used for all the samples during image acquisition. Picture intensity was quantified using ImageJ and the average of the intensities of all the acquired fields was considered for each sample. Arginase-1 and iNOS intensity for each image was normalized for the DAPI channel intensity and expressed as relative intensity, as previously described^21^.

### Corrected TIMI frame count (CTFC)

CTFC was calculated with the angiogram images (TOSHIBA DICOM viewer).

### *Ex vivo* flux assay

Campos JC *et al*. recently published a protocol (Scientific Reports 2018) where autophagic flux is measured *ex vivo* in skeletal muscle^22^. We modified this protocol regarding dosage and timing of chloroquine treatment in order to optimize the latter for myocardial tissue, which has not been described before. Essentially, a specimen is divided immediately after harvest into fine pieces equally divided into two groups (with and without chloroquine treatment). After a certain incubation time, the tissue is extracted and snap frozen for further analysis of autophagy markers.

### Mitochondrial respirometry

Respirometry on isolated mitochondria was performed with a Seahorse XF Analyzer (Agilent Technologies, Santa Clara, CA, USA) as previously described^23^. We used XFp plates loading 2.5 µg of protein/well. Port A was loaded with 2.5 mM ADP, 100 mM Pyruvate and 20 mM Malate. Ports B-D were loaded with 20 µM Oligomycin, 20 µM Carbonyl cyanide 4-(trifluoromethoxy)phenylhydrazone (FCCP) and 10 µM AntimycinA/Rotenone, respectively. Data analysis was performed with WAVE (Agilent Technologies, Santa Clara, CA, USA).

### Human tissue collection

Patients with severe reduction of ejection fraction and heart failure eligible for left ventricular assist device (LVAD), total artificial heart (TAH) implantation or heart transplantation (HTx) were asked by the cardiology department physicians for consent to transfer discarded heart tissue to the tissue repository at Cedars Sinai (IRB Protocol number Pro11910). This tissue is made available to investigators after IRB approval of the proposed use of tissue. Informed consent was obtained from all patients included in the study. For the purpose of our laboratory we collect heart tissue from patients with heart failure with severe reduction of left ventricular ejection fraction scheduled for LVAD, TAH or HTx after previous assist device implantation, as only inclusion criteria, independent of any other patient characteristics. From consenting patients, we collected 0.5 cm^3^ of apical specimens of the LV during LVAD implantation, as approved by the Institutional Review Board (IRB) of CSMC (Protocol number Pro38966). All samples were collected and stored in accordance with the Guidelines for Human Tissue Repository of the NIH (National Heart, Lung and Blood Institute). The samples were collected during surgery, immediately after the LVAD coring device was inserted into the apex of the LV, which leads to the excision of a cylindrical, transmural piece of myocardium. The specimen was immediately placed on ice and snap frozen for further processing. Retrospectively, we reviewed the surgical records to obtain the core temperature registered during surgery. This enabled us to use human tissue for this study and evaluate whether our findings were clinically translatable. For this study we grouped patients into those with a registered core temperature below 34 °C, or above 36 °C, corresponding to the temperature ranges achieved in our pig model. Core temperature was the only inclusion criteria for our study independent of all other patient characteristics (Supplemental Fig. 4). Although the cooling period during surgery is unique to each patient, given the duration of the procedure we are confident that the 60 minute cooling in our pig model is comparable to the time used during surgery.

### Statistical analysis

All quantitative data analysis of this study corresponds to the mean ± SEM. WB, blood markers, mRNA and DNA, echocardiography, temperature and CTFC analysis were done using a parametric *t*-test. Autophagy flux and lymphocyte proliferation were evaluated by 2-way analysis of variance (ANOVA, GraphPad Prism 6.) Arginase-1, iNOS, apoptosis and Picrosirius red staining was quantified through non-parametric *t*-tests (Mann-Whitney). Statistical significance was obtained with a p-value < 0.05.

Given the nature of the data, being continuous and coherent among each other, without significant outliers, we used parametric statistics for most of our experiments. The significant p-values for many of the results, were also complemented by trends that were consistent with the primary findings. Nonetheless, we are aware that with a low sample size, non-parametric statistics strengthen the data; therefore, we performed a control with Mann-Whitney test on most of our significant result in order to ensure the correctness of the data, leading to the same conclusion.

## Results

### Reduction of no-reflow and ischemic tissue damage

**Figure 2:**
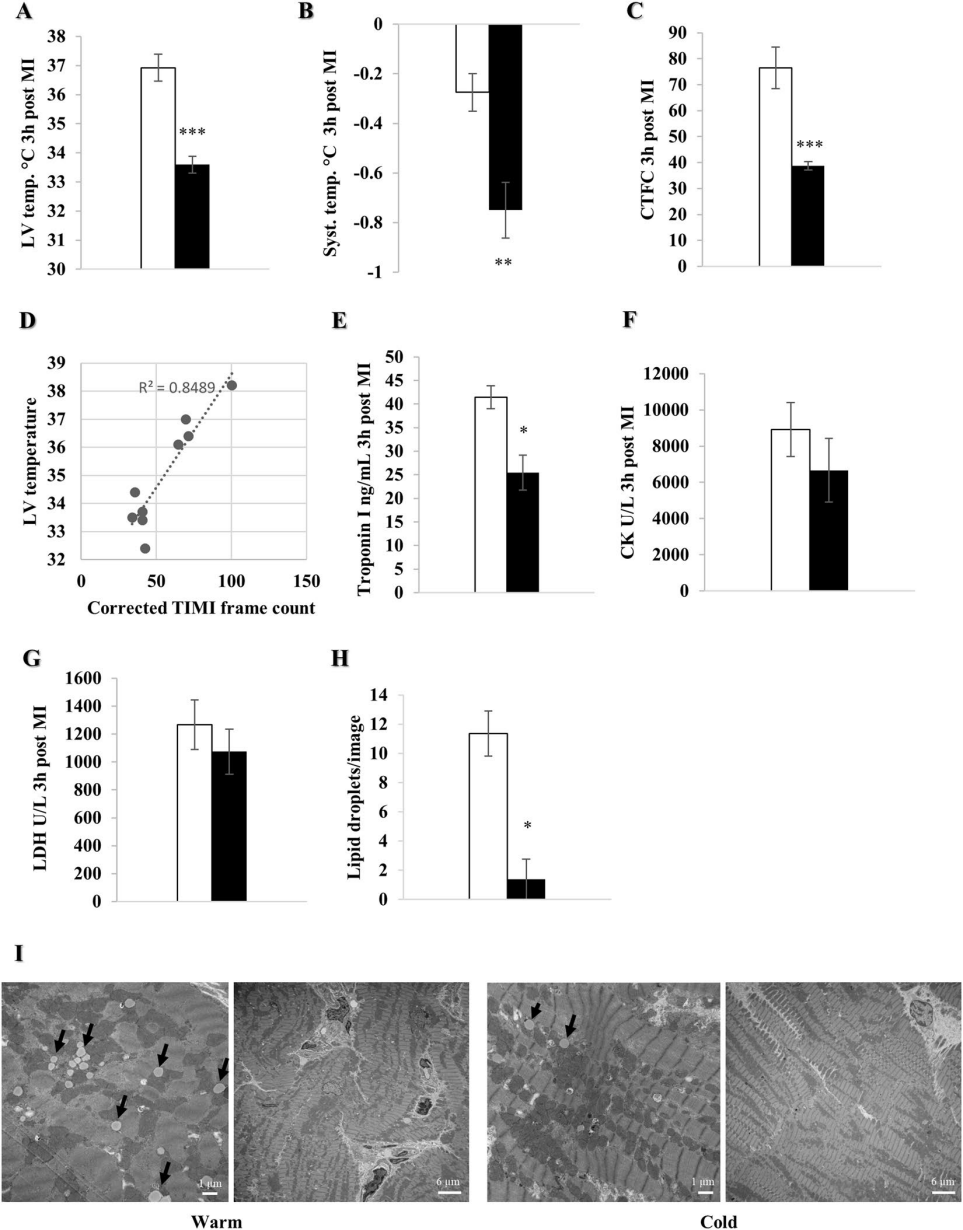
Local pericardial cooling reduces no-reflow and ischemic tissue damage. Female farm pigs were divided in warm (white) and cold (black) and underwent 90 min of MI, followed by reperfusion and 60 min of pericardial irrigation. (**A**,**B**) LV and systemic temperature measurement respectively (n = 6 and n = 4 in the cold and warm group respectively), (**C**) CTFC calculation (n = 5 and n = 4 in the cold and warm group respectively), (**D**) linear correlation between the CTFC and LV temperature (n = 5 and n = 4 in the cold and warm group respectively). (**E**–**G**) Release of serum cardiac troponin I (ng/mL), creatinine kinase (U/L) and lactate dehydrogenase (U/L) respectively (n = 3 per group). (**H**) Quantification of neutral lipid droplets (n = 4 randomly chosen images/animal). (**I**) Representative EM images (x2500 and x10000 respectively for each group) of lipid droplets from LV septal biopsies (black arrows: neutral lipid droplet). All data were obtained 3 h post MI and are represented by the mean ± SEM with *p < 0.05, **p < 0.01, ***p < 0.001 comparing cold vs. warm.

To assess the efficacy of local pericardial cooling we measured LV and systemic temperature 3 h after MI. Pericardial irrigation with cold saline lowered LV temperature (33.6 ± 0.3 °C vs. 36.9 ± 0.5 °C, p < 0.001), which was accompanied by a drop in systemic temperature (p < 0.01) (Fig. 2A,B). Efficacy of TH was demonstrated by a significantly decreased Corrected Thrombolysis in Myocardial Infarction (TIMI) frame count (CTFC) (p < 0.001) and a very strong linear correlation (R^2^ = 0.85) between LV temperature and CTFC in the cold (p < 0.001) (Fig. 2C,D). Blood analysis 3 h post MI showed a significant reduction of cardiac troponin I (p < 0.05); consistent with that, creatinine kinase and lactate dehydrogenase also trended lower in the cold (Fig. 2E–G). Lastly, EM images of LV septal biopsies 3 h post MI showed a significant reduction of neutral lipid droplets (p < 0.05) correlating with tissue injury (Fig. 2H,I)^23^.

### Improvement of myocardial function four weeks post MI

**Figure 3:**
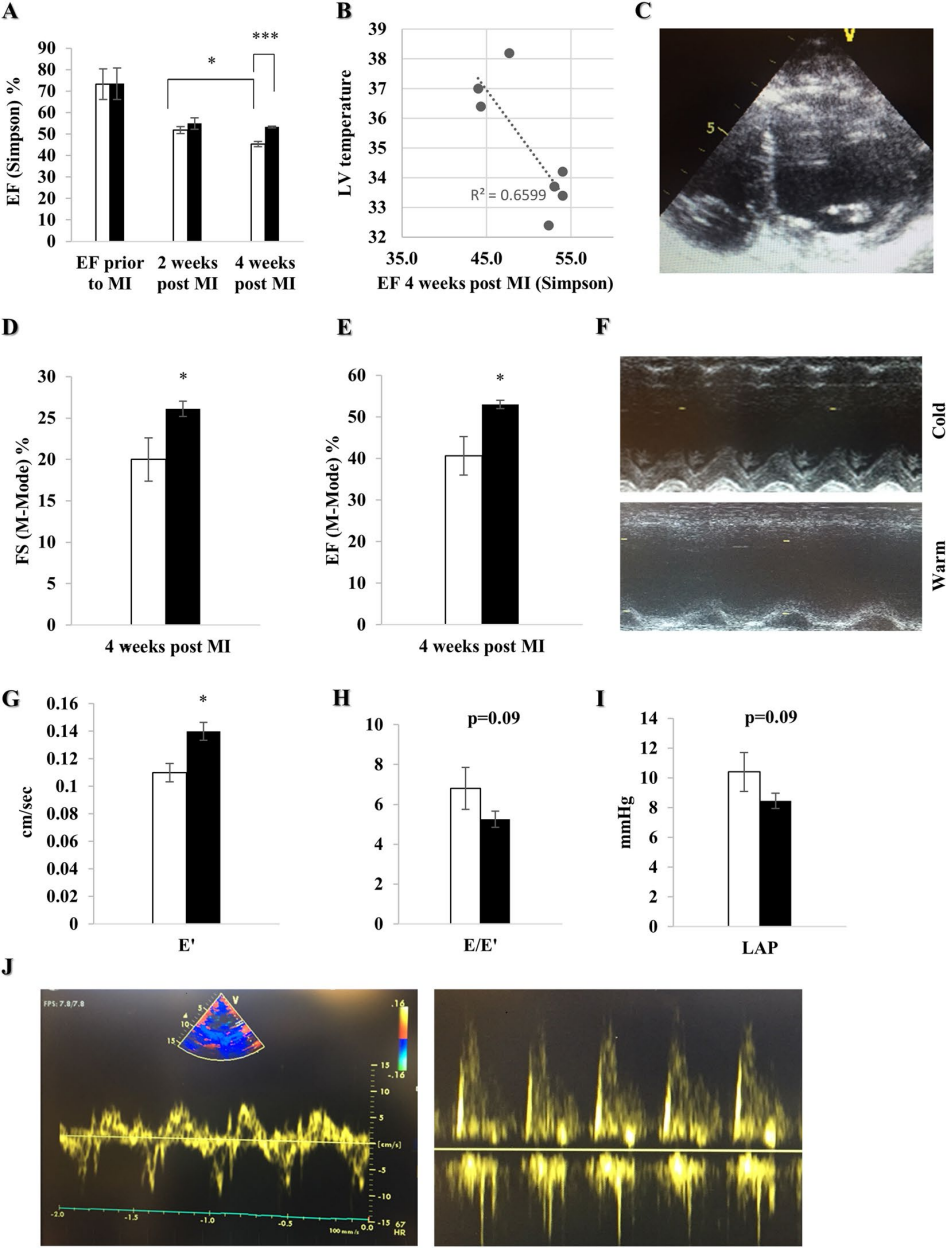
Local pericardial cooling improves myocardial function four weeks post MI. Transthoracic echocardiography was performed prior to, two and four weeks post MI in female farm pigs divided in a warm (n = 3, white) and cold (n = 4, black) group. (**A**) EF measured by Simpson method prior to MI, two and four weeks post MI. (**B**) Linear correlation between EF (Simpson method) four weeks post MI and LV temperature. (**C**) Four-chamber view of the LV used for the Simpson method. (**D**,**E**) FS and EF obtained in M-Mode, with the Teichholz formula, four weeks post MI. (**F**) M-Mode image in the parasternal long axis through the LV in both groups four weeks post MI. (**G**–**I**) Evaluation of diastolic function by measuring E’(cm/sec), E/E’ ratio and calculated LAP (mmHg) respectively, using the combination of PW and TVI four weeks post MI. (**J**) Illustration of PW and TVI respectively. The data represents the mean ± SEM with *p < 0.05, ***p < 0.001 comparing cold vs. warm.

To investigate the effect of local hypothermia on remodeling and myocardial function, we performed transthoracic echocardiography (Simpson method) prior to, two and four weeks after MI. There was no difference between the groups prior to MI. Although there is no significant difference in EF two weeks after MI in the cold compared to the warm group, we observed a significant decline of EF between weeks two and four post MI in the warm group (p < 0.05), whereas the EF in the cold group during this timeframe remained rather stable. Furthermore, 4 weeks after MI and cold irrigation, EF was significantly increased when compared to warm (p < 0.001), with a strong correlation (R^2^ = 0.66) between LV temperature and EF (p = 0.03) (Fig. 3A,B). The assessments were repeated with the Teichholz method, showing a significantly reduced EF (p < 0.05) and FS (p < 0.05) in the warm compared to the cold four weeks post MI (Fig. 3D–F). Additionally, we found a significantly increased E’(p < 0.05), as well as a trend towards reduced E/E’(p = 0.09) and LAP (p = 0.09) in the cold, suggesting improved diastolic function (Fig. 3G–J).

### Increase in autophagic flux

**Figure 4:**
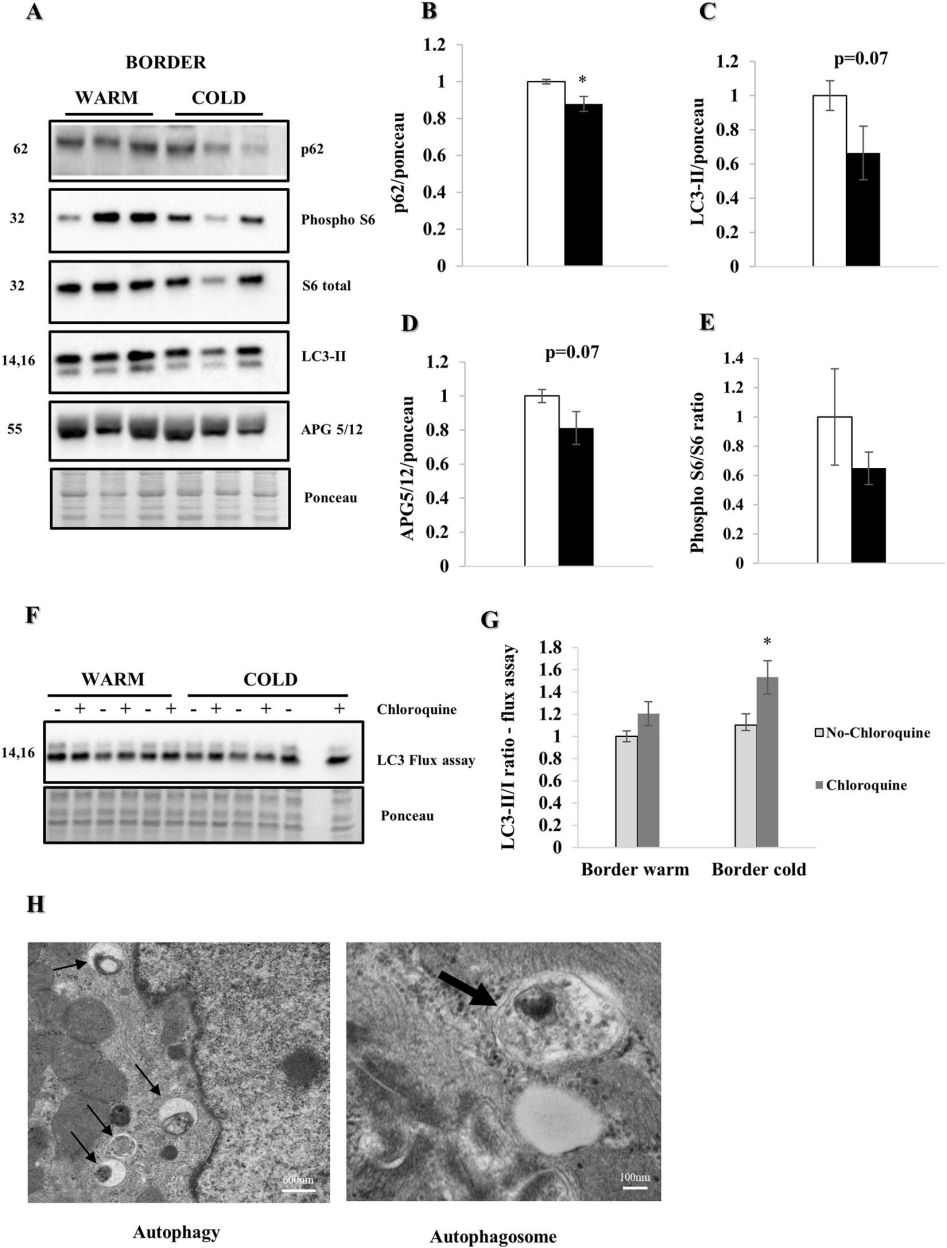
Myocardial hypothermia increases autophagic flux. Female farm pigs (n = 3 per group) were euthanized one week post MI for biochemistry analysis on whole lysate of the LV border zone. All proteins were normalized to ponceau. (**A**) WB analysis with protein quantification (**B**–**E**) of p62, LC3-II APG5/12 and phospho-S6/S6 (Ser 235/236) ratio respectively. (**F**) WB analysis and quantification (**G**) of LC3-II/I ratio of the *ex vivo* autophagy flux assay (light gray bars with black border represent tissue without chloroquine, dark grey bars tissue with chloroquine treatment respectively). The data represents the mean ± SEM with *p < 0.05, comparing cold (black) vs. warm (white). (**H**) Representative EM images (3 h post MI, x30000 and x120000 respectively; the black thin arrows in the left image, show multiple autophagosomes due to enhanced autophagy in the cold condition with zoom on a single autophagosome in the right image). Due to space limitation, the blots are cropped from various gels, please see the supplemental data for full-length gels/blots.

WB analysis (Fig. 4A) one week post MI showed a significant reduction of p62 in the cold compared to the warm (p < 0.05) (Fig. 4B). Additionally, we found a non-significant reduction of LC3-II (p = 0.07) and APG5/12 (p = 0.07) in the cold vs. warm, suggesting increased autophagy. Furthermore, the trend towards a decrease of phospho-S6/S6 (Ser 235/236) ratio supports our hypothesis of enhanced autophagy, with a decreased mTOR pathway activity being one possible mechanism of action (Fig. 4C–E). An *ex vivo* autophagy flux assay with chloroquine confirmed a significant increase in autophagic flux (p < 0.05) in the cold group (Fig. 4F,G). EM images (Fig. 4H) demonstrate multiple autophagosomes consistent with increased autophagy after cold irrigation.

### Increase in mitophagy

**Figure 5:**
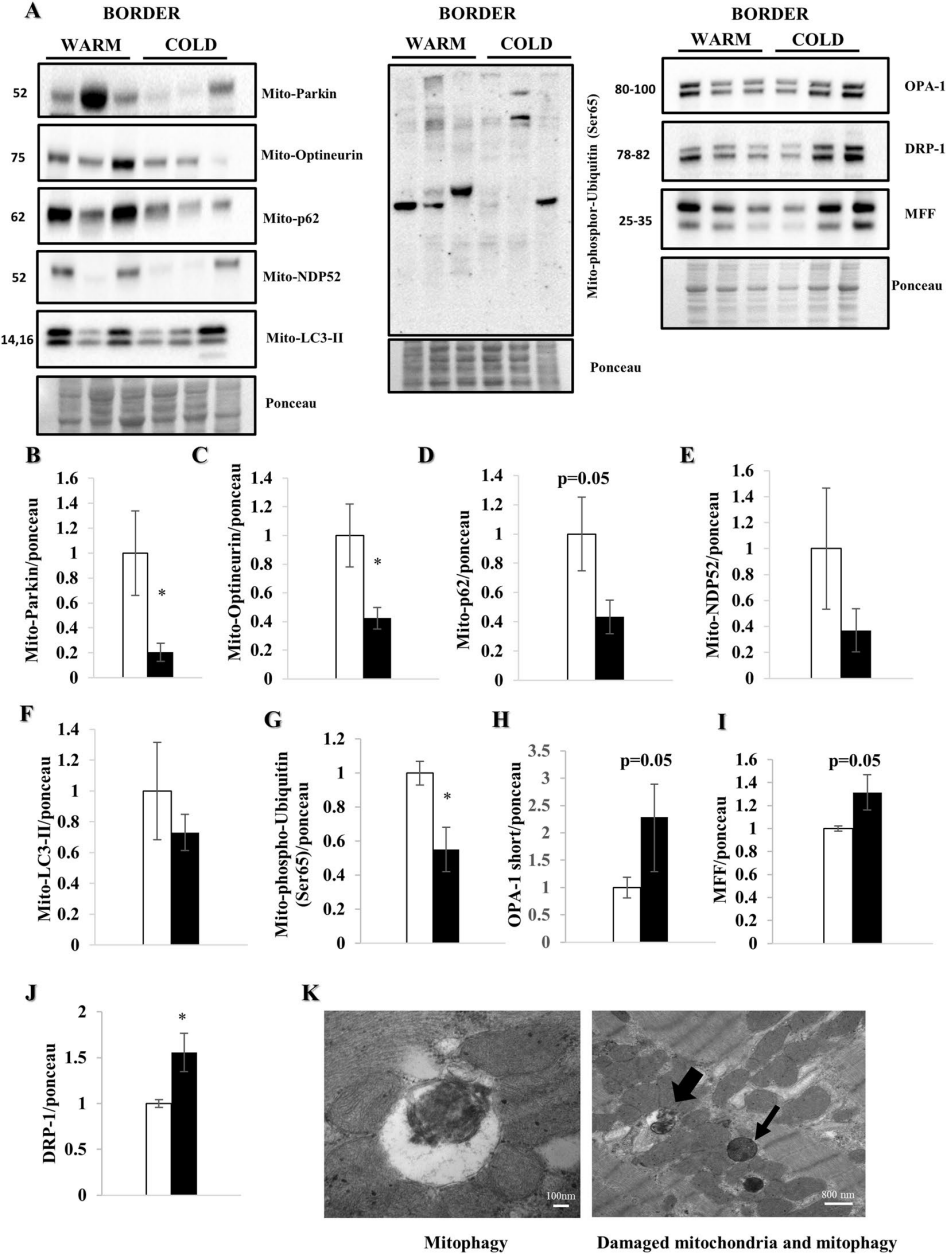
Myocardial hypothermia increases mitophagy. Female farm pigs (n = 3 per group) were euthanized one week post MI. WB analysis were performed on the mitochondrial fraction of myocardial tissue. All proteins were normalized to ponceau. (**A**) WB analysis with protein quantification (**B**–**G**) of parkin, Optineurin, p62, NDP52, LC3-II and phospho-Ubiquitin (Ser65) respectively in the mitochondrial fraction. Our hypothesis of mitophagy is supported by a strong increase of the short form of OPA-1 and MFF, as well as a significant increase of DRP-1 in the whole lysate (**H**–**J**) The data represents the mean ± SEM with *p < 0.05, comparing cold (black) vs. warm (white). (**K**) Representative EM images (3 h post MI) of) mitophagy (x100000) and damaged mitochondria (thin black arrow) with mitophagy (thick black arrow) (x19000) under cold condition. We normalized each protein to the entire corresponding lane on the ponceau-stained membrane, representing the total protein amount. Due to space limitation, the blots are cropped from various gels, please see the supplemental data for full-length gels/blots.

To further investigate the mechanisms of cell recovery under TH, we focused on proteins involved in mitophagy (Fig. 5A). TH significantly reduced parkin (p < 0.05) and optineurin (p < 0.05) in the mitochondrial fraction (Fig. 5B,C). Additionally, p62 (p = 0.05), NDP52 and LC3-II in the heavy membrane (mitochondria-enriched) fraction were lower, although this did not reach statistical significance (Fig. 5D–F). These results are supported by significantly decreased phospho-ubiquitin (Ser65)-positive mitochondria (p < 0.05) (Fig. 4G). We interpret these results to indicate increased clearance of damaged mitochondria after cold exposure, compared with ongoing mitochondrial damage and mitophagy in the warm hearts. Furthermore, we analyzed fission regulators DRP-1, MFF and OPA-1 (short and long forms) in the whole lysate. DRP-1showed a significant increase under cold irrigation, while OPA1 and MFF trended towards increasing (Fig. 5H–J). These findings suggest increased mitochondrial fission, a process that accompanies mitophagy. Figure 5K shows micrographs of a cold-irrigated heart 3 hours post MI, demonstrating damaged mitochondria in tissue and as cargo in autophagosomes.

### Increased mitochondrial mass and function

**Figure 6:**
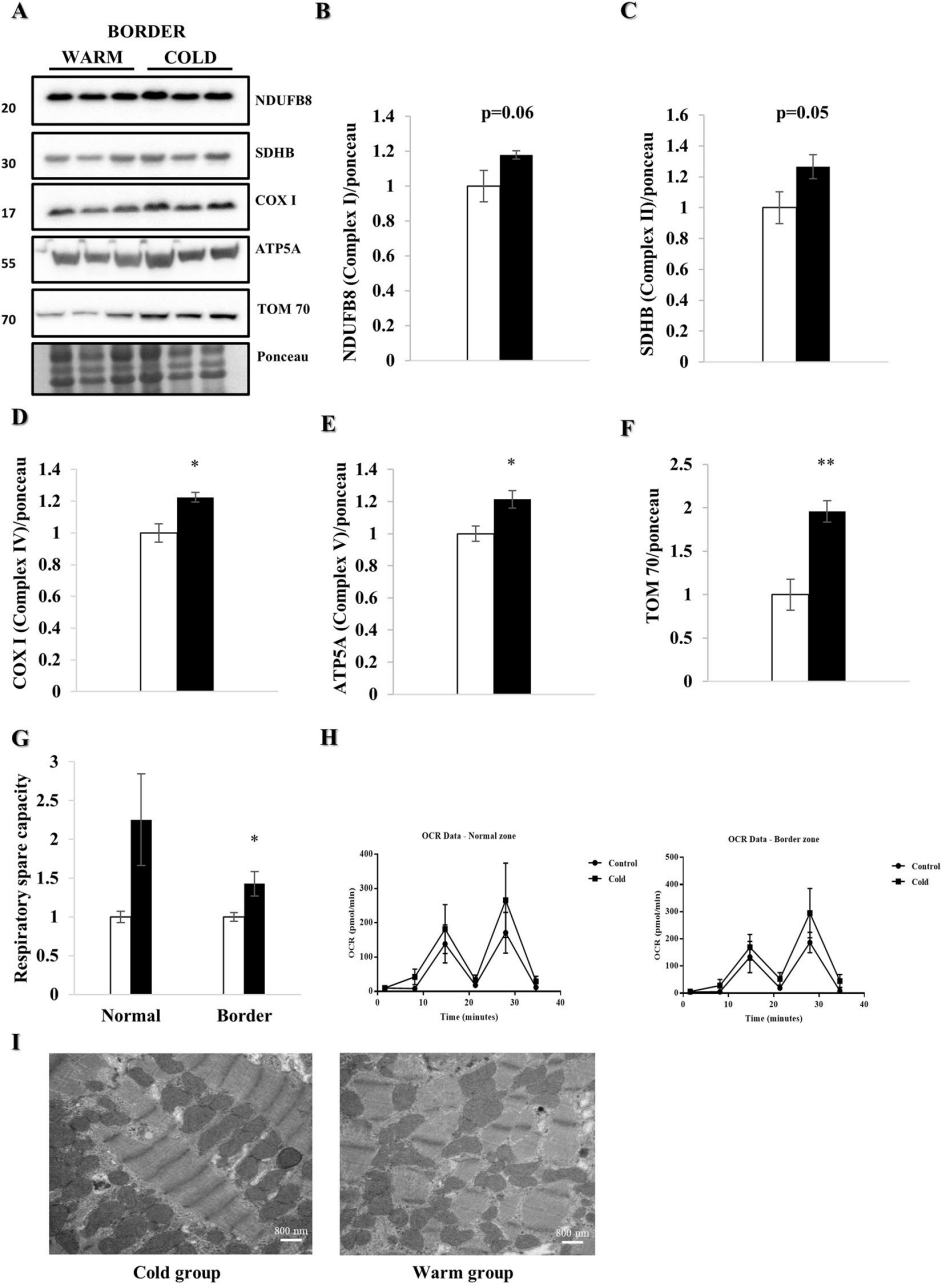
Mitochondrial mass and function are increased under hypothermia. LV border zone tissue from female farm pigs (n = 3 per group) was harvested one week post MI. WB analyses were performed on the whole lysate. All proteins were normalized to ponceau. (**A**) WB analysis with protein quantification (**B**–**F**) of NDUFB8, SDHB, COXI, ATP5A and TOM70 respectively. Mitochondrial respirometry analyses were performed on fresh isolated mitochondria of LV tissue (n = 7) 4 weeks post MI. (**G**) Respiratory spare capacity and respirometry trace (**H**) of the normal and border zone in the warm and cold group. (**I**) Representative EM images of mitochondria (x19000) demonstrating a disruption of density and structure of mitochondria in the warm condition after MI compared to the cold. The data represents the mean ± SEM with *p < 0.05, **p < 0.01, comparing cold (black) vs. warm (white). We normalized each protein to the entire corresponding lane on the ponceau-stained membrane, representing the total protein amount. Due to space limitation, the blots are cropped from various gels, please see the supplemental data for full-length gels/blots.

To assess mitochondrial content, we focused on proteins of the oxidative phosphorylation machinery (Fig. 6A). NADH dehydrogenase 1-beta subcomplex subunit 8 (NDUFB8, of complex I, p = 0.06), Succinate dehydrogenase iron-sulfur subunit (SDHB, of complex II, p = 0.05), Cytochrome *c* oxidase subunit I (COXI, of complex IV, p < 0.05) and ATP synthase subunit alpha (ATP5A, of complex V, p < 0.05) were increased in the cold-exposed group (Fig. 6B–E), as was TOM70 (p < 0.01), reflecting increased mitochondrial protein mass one week after MI and cold irrigation (Fig. 6F). As cardiac contractility depends upon adequate mitochondrial ATP production, we performed mitochondrial respirometry four weeks post MI. Therefore, we isolated mitochondria from fresh tissue and calculated the protein concentration. We subsequently added 1.5 μg of mitochondrial protein in each well of a XFp Seahorse plate. Even after correcting for increased mitochondrial protein content in the cold-exposed hearts with total protein amount, respiratory spare capacity was significantly increased (p < 0.05), suggesting more oxidative phosphorylation machinery per mitochondrion (Fig. 6G,H). Interestingly, as seen in the EM images of Fig. 6I, it seems as if mitochondrial structure and density 3 hours after I/R injury are more completely restored after cold irrigation compared to the warm group.

### Decreased myocardial cell stress

**Figure 7:**
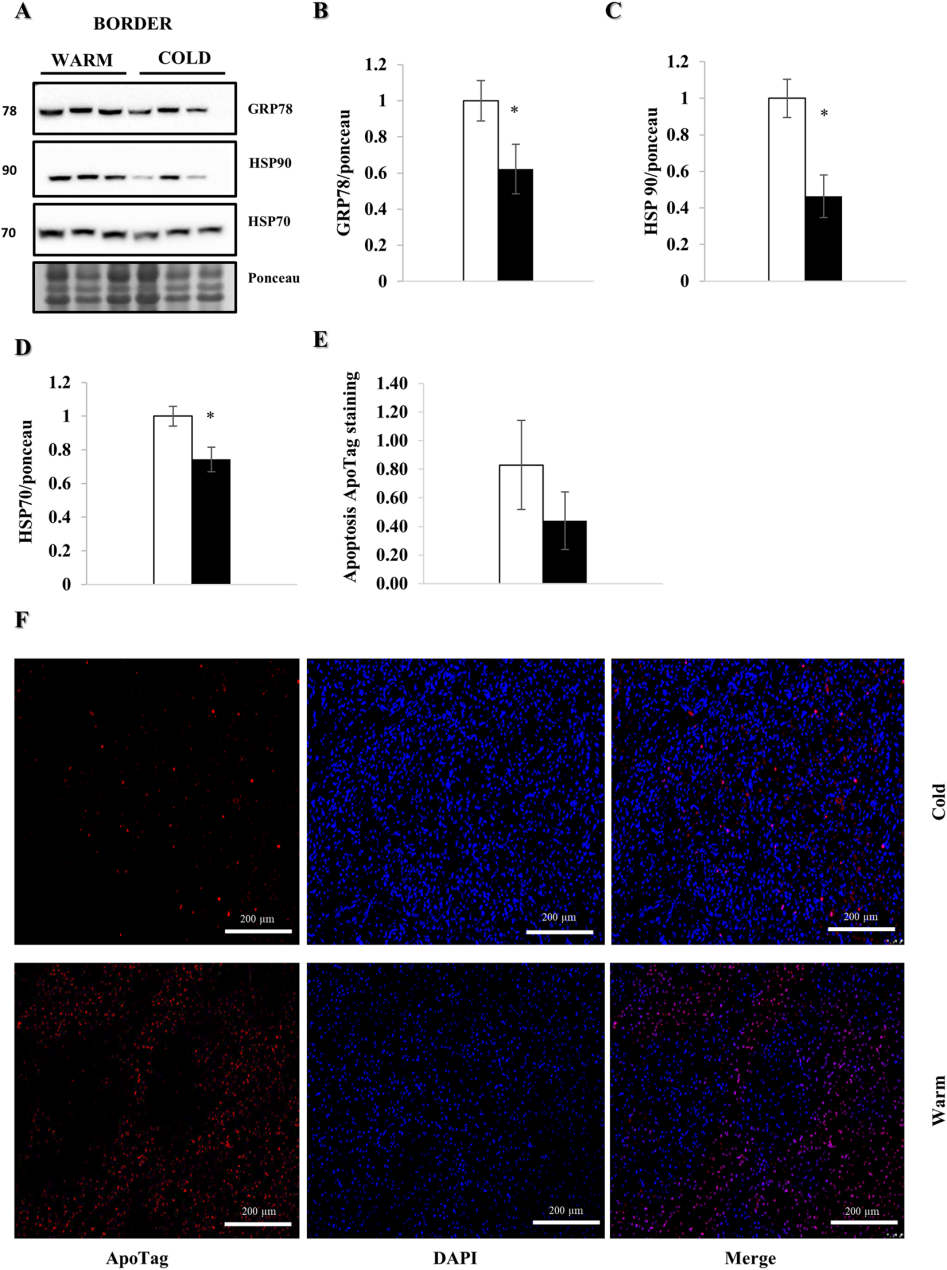
Myocardial hypothermia decreases cell stress. Myocardial tissue from female farm pigs (n = 3 per group) was harvested one week post MI. WB analysis were performed on the whole lysate of the LV border zone. All proteins were normalized to ponceau. (**A**) WB analysis with protein quantification (**B**–**D**) of GRF78, HSP90 and HSP70 respectively. (**E**) Quantification of apoptosis staining with ApoTag in the myocardium (n = 6). (**F**) Representative images of apoptotic cells detected with the ApoTag staining kit (x10). The data represents the mean ± SEM with *p < 0.05, comparing cold (black) vs. warm (white). We normalized each protein to the entire corresponding lane on the ponceau-stained membrane, representing the total protein amount. Due to space limitation, the blots are cropped from various gels, please see the supplemental data for full-length gels/blots.

WB analysis one week post MI showed a significant reduction of cellular stress markers GRP78 (p < 0.05), HSP90 (p < 0.05) and HSP70 (p < 0.05) in the cold (Fig. 7A–D). Additionally, we found a 47% reduction in the number of apoptotic cells in the cold compared to the warm, although this did not reach statistical significance (Fig. 7E,F).

### Decreased local and systemic inflammation

**Figure 8:**
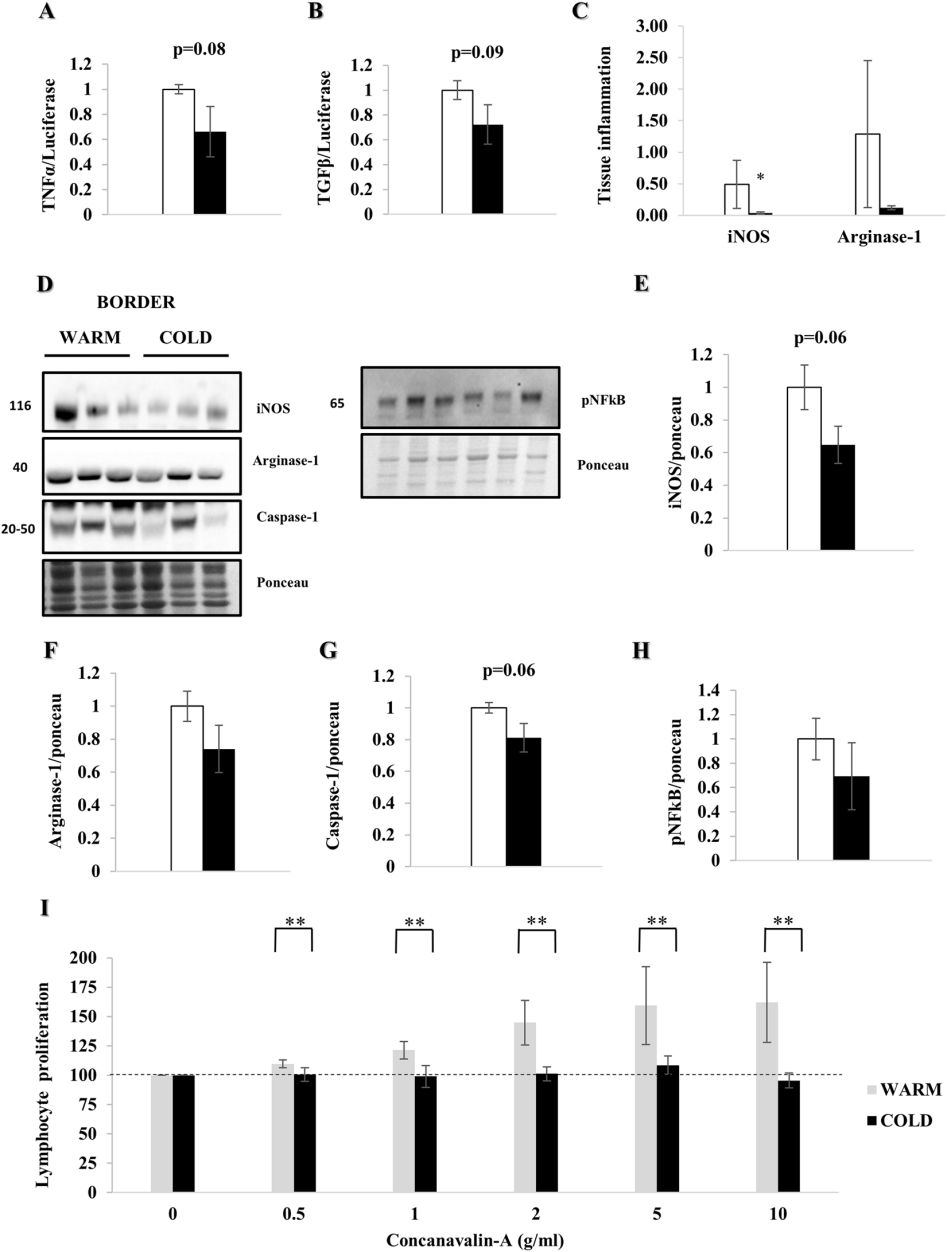
Myocardial hypothermia decreases local and systemic inflammation. Myocardial border zone tissue of female farm pigs (n = 3 per group) was harvested one week post MI. (**A**,**B**) Quantification of TNFα and TGFβ mRNA expression respectively. (**C**) Quantification of local tissue inflammation via iNOS and Arginase-1 staining (representative images: Supplemental Fig. 2). (**D**) WB analysis with protein quantification (**E**–**G**) of iNOS, Arginase-1 and Caspase-1 respectively in whole lysate and (**H**) pNFκB in the nuclear fraction. (**I**) Quantification of lymphocyte proliferation with Concanavalin-A treatment on fresh splenic tissue, harvested from the same pigs. The data represents the mean ± SEM with *p < 0.05, **p < 0.01, comparing cold (black) vs. warm (white). We normalized each protein to the entire corresponding lane on the ponceau-stained membrane, representing the total protein amount. Due to space limitation, the blots are cropped from various gels, please see the supplemental data for full-length gels/blots.

One week after MI, mRNA expression of TNFα (p = 0.08) and TGFβ (p = 0.09) trended lower in the cold (Fig. 8A,B). Furthermore, immunostaining demonstrated a significant reduction of iNOS (p < 0.05) and Arginase-1, indicating decreased local tissue inflammation (Fig. 8C, Supplemental Fig. 2). WB confirmed a non-significant reduction of iNOS, Arginase-1 and Caspase-1 (p = 0.06) (Fig. 7E–G), as well as pNFκB in the nuclear fraction (Fig. 8H). Additionally, lymphocyte proliferation in response to concanavalin-A was attenuated in the cold-exposed animals (p < 0.01), consistent with reduced systemic inflammation (Fig. 8I).

### Decrease in myocardial fibrosis

**Figure 9:**
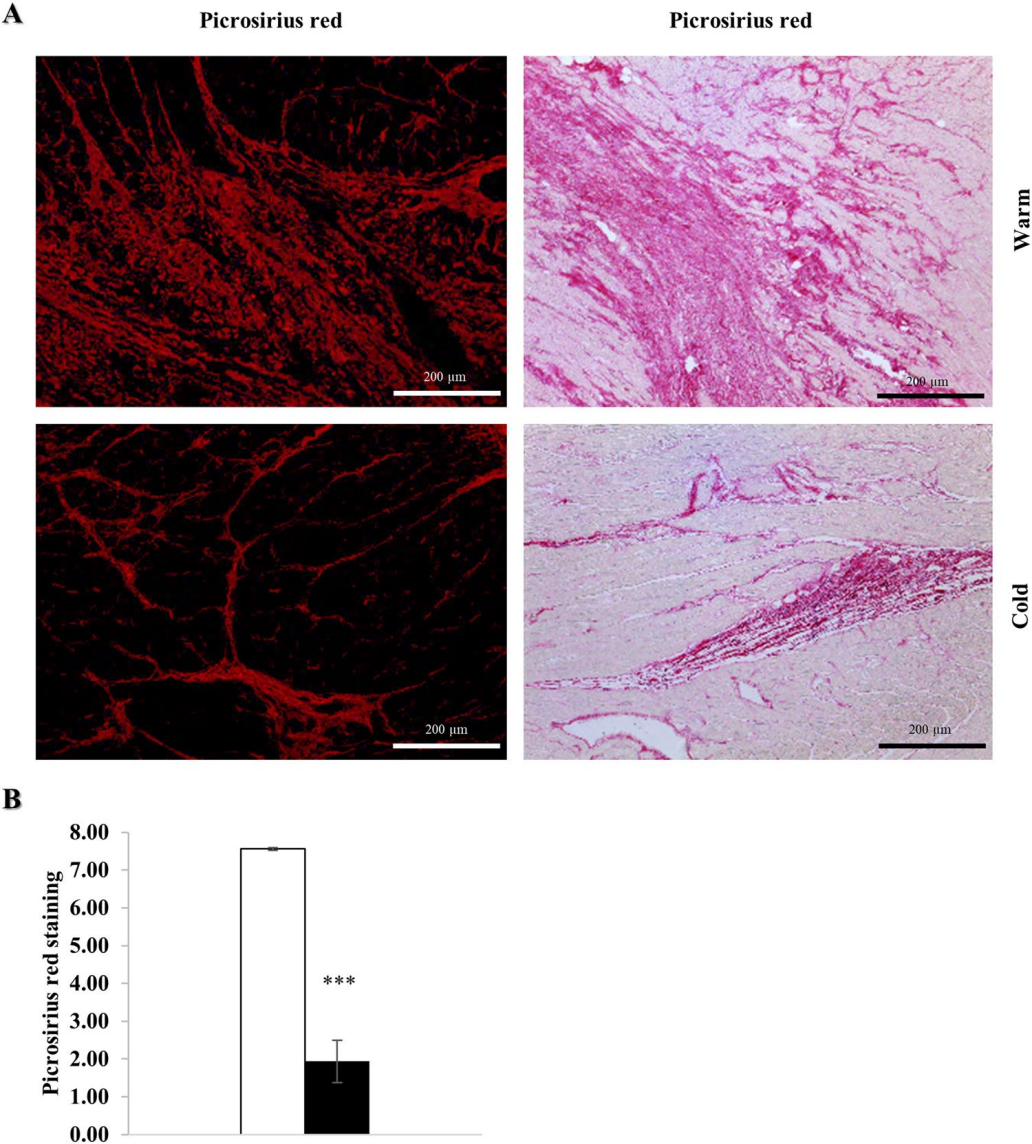
Myocardial hypothermia decreases fibrosis. Myocardial tissue of the LV border zone from female farm pigs (n = 3 per group) was harvested one week post MI and subsequently fixed in 4% Formaldehyde for histology. (**A**) Picrosirius red staining with fluorescence and optical microscope respectively. (**B**) Quantification of fibrosis measured with Picrosirius red. The data represents the mean ± SEM with ***p < 0.001, comparing cold (black) vs. warm (white).

One week after MI, myocardial tissue was harvested and stained with Picrosirius red for quantification of fibrosis, which was significantly decreased in the cold (p < 0.001) (Fig. 9).

### Increased autophagy and mitophagy in cardiac surgery under hypothermic conditions

**Figure 10:**
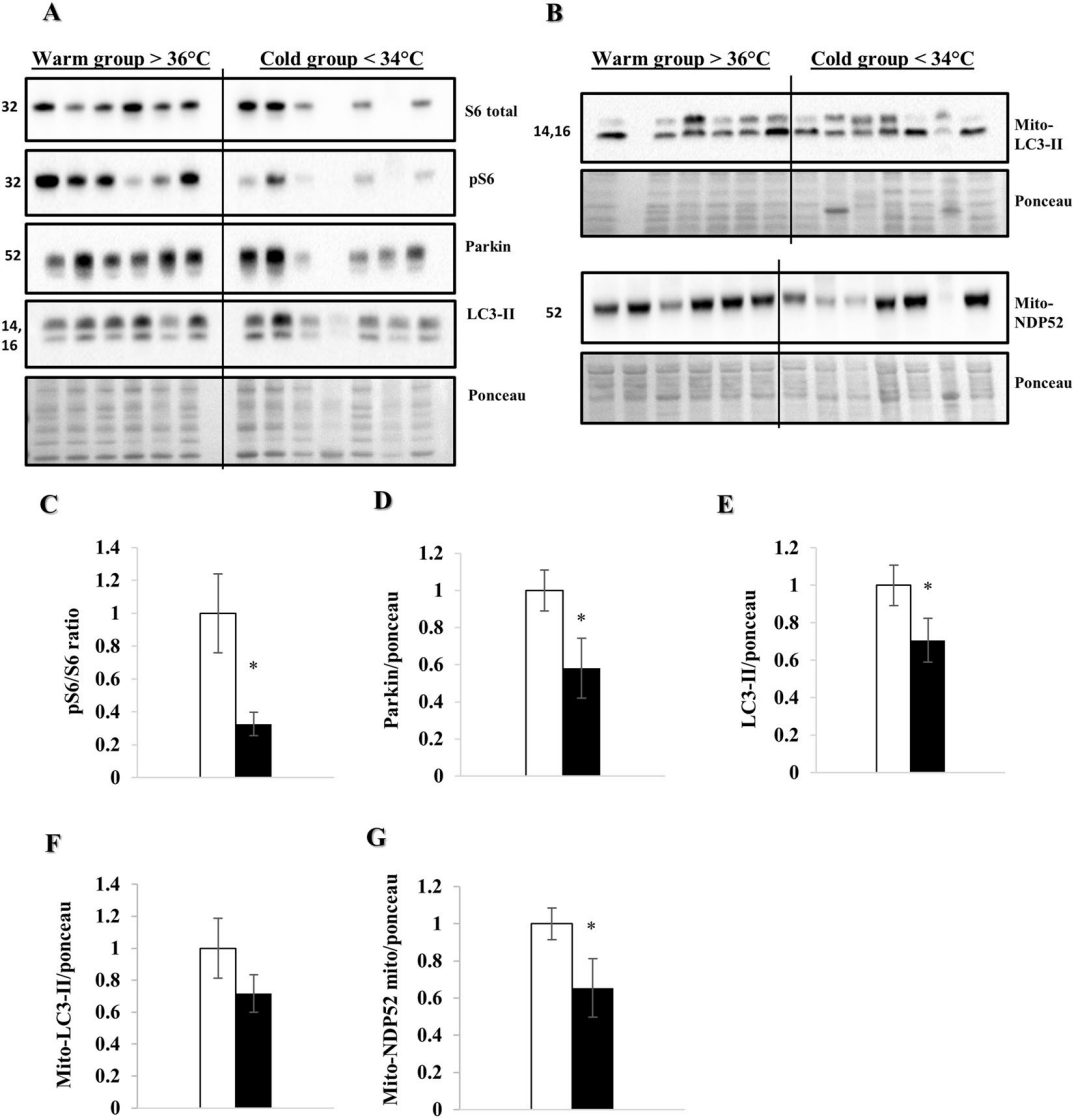
Autophagy and mitophagy are increased during cardiac surgery in a hypothermic setting. WB analyses were performed on LV apical tissue obtained during LVAD procedure (n = 6 and n = 7 in the warm and cold group respectively). (**A**,**B**) WB analysis on whole lysate and mitochondrial fraction respectively. Protein quantification (**C**–**E**) of parkin, phospho-S6/S6 ratio and LC3-II in the whole lysate, (**F**,**G**) LC3-II and NDP52 in the mitochondrial fraction respectively. All proteins were normalized to ponceau. The data represents the mean ± SEM with *p < 0.05, comparing cold (black) vs. warm (white). We normalized each protein to the entire corresponding lane on the ponceau-stained membrane, representing the total protein amount. Due to space limitation, the blots are cropped from various gels, please see the supplemental data for full-length gels/blots.

To evaluate whether hypothermia induces similar responses in human heart tissue, we performed WB analysis on apical tissue obtained during LVAD implantation (Fig. 10A,B). We compared patients with a registered temperature below 34 °C to those above 36 °C during surgery, equivalent to our pig study. pS6/S6 ratio (p < 0.05), parkin (p < 0.05) and LC3-II (p < 0.05) were significantly reduced in the cold group (Fig. 10C–E). Similarly, LC3-II trended and NDP52 significantly (p < 0.05) decreased in the heavy membrane fraction in the cold (Fig. 10F,G), which we interpret to reflect increased autophagic/mitophagic flux, consistent with our animal data.

## Discussion

The beneficial effects of TH on cardiomyocyte function, MI size, no-reflow and remodeling demonstrated in animal models are incontestable^2–5^, yet TH has not demonstrated clear benefits in the clinical setting of acute MI. Previous studies mainly focused on reduction of MI size and no-reflow, initiating TH either before or immediately after coronary occlusion, which may be impractical in the clinical setting^2–5,8^. To our knowledge this is the first study focusing on treatment with TH after reperfusion with long time survival. The clinically translatable time course and the specific aim of deciphering molecular mechanisms impacting remodeling, distinguishes our model from others.

With a local approach of pericardial cooling, we achieved the same impact on the myocardium as did other systemic methods^5^, shown by significant reduction of LV and systemic temperature. Furthermore, the significant decrease of Troponin I, CTFC and strong correlation between LV temperature and CTFC substantiate the reduction of tissue damage, its beneficial effects on the myocardium and therefore the efficacy of this model^8,9,24,25^. This is confirmed by a significant reduction of lipid droplets in the cold. Lipid droplets accumulate in injured cells due to decreased fatty acid β-oxidation, associated with transiently increased mitochondrial membrane potential and ROS production^24^. Echocardiography data clearly showed that TH attenuated EF decline between two and four weeks post MI, with significantly better preservation of EF and FS four weeks post MI. In addition, diastolic function showed a trend towards improvement, although LAP did not reach statistical significance. Consistent with this, we observed less fibrosis in cold-exposed hearts seven days post MI. We therefore focused on investigating potential mechanisms involved in remodeling.

The significant reduction of established autophagy marker p62, and a trend for reduced LC3-II and APG5/12 suggest an increase in autophagic flux. This is supported by a significant increase in autophagic flux measured by *ex vivo* flux assay. We established the latter in our lab as an easily reproducible assay, validated in heart tissue of various species (mouse, pig and humans). The aim of the assay is to facilitate analysis of autophagy markers and verify whether protein analysis by WB of autophagy markers such as LC3-II/I ratio is related to increased autophagic flux. Static WB analysis of those markers can be difficult and misleading under certain circumstances. The assay is currently under submission for publication. Reduced autophagy is associated with increased remodeling and tissue damage^13,16,17^.

Specific mitophagy markers such as parkin and optineurin were significantly reduced in the mitochondrial fraction. Consistent with this p62, NDP52 and LC3-II also trended lower in the hearts exposed to cold, suggesting increased clearance of damaged mitochondria^26–28^. Additionally, we noted a decrease in phospho-ubiquitin (Ser65)-positive mitochondria suggestive of increased clearance of damaged mitochondria under cold conditions. Fission regulators DRP-1, MFF and the short form of OPA-1 increased after cold irrigation; as fission is a prerequisite of mitophagy, these changes are supportive of our interpretation that cold exposure resulted in enhanced mitophagy even seven days post MI. Furthermore, the lower phospho-S6/S6 (Ser 235/236) ratio, although not statistically significant given the modest number of animals, suggests a decrease of the mechanistic target of Rapamycin (mTOR) pathway activity as one possible regulatory pathway^16,27^. It is remarkable that a brief (60 min) episode of hypothermia initiated 30 minutes after onset of reperfusion could elicit sustained changes in autophagy/mitophagy, with improved mitochondrial function in the border zone and better preservation of cardiac contractility at fourteen days post MI. Mitochondria regulate calcium signaling, membrane potential, cellular metabolism, apoptosis, as well as ATP and ROS production and their adequate function is essential for myocardial performance. Dysfunctional mitophagy has been associated with various heart diseases as well as adverse remodeling and HF^10–12,14,15^. The significant increase in autophagic flux and mitophagy in the cold, therefore suggest more efficient cell renewal^11–16^. Additionally, we observed a significant increase in some of the proteins of oxidative phosphorylation (NDUFB8, SDHB, COX IV and ATP5A) as well as TOM70, suggesting increased mitochondrial machinery after cold exposure^15,19^. This data is consistent with a study of Andres *et al*. showing similar protein increases suggesting biogenesis^19^, although they observed an increase in mitochondrial DNA, which we did not. However, the Andres study examined atrial biopsies at the end of cardiopulmonary bypass whereas this study examined mitochondrial content seven days post MI. It is possible to argue that we are detecting accumulation of mitochondrial proteins due to impaired degradation. To address this, we performed qPCR for mRNA quantification of TFAM, NRF 1 and NRF 2 (Supplemental Fig. 4). We did not detect a statistically significant difference in mRNA expression at seven days post MI; thus, we do not have clear evidence for or against mitochondrial biogenesis. Additional studies will be required.

Consistent with the increase in mitochondrial mass, respirometry of isolated mitochondria demonstrated increased respiratory spare capacity, suggestive of higher capacity for ATP production to support contraction. The latter is consistent with our finding of increased LVF four weeks after MI after cold exposure. Taken together, the evidence presented herein describe increased autophagy/mitophagy after cold exposure, which we hypothesize leads to mitochondrial turnover, resulting in increased mitochondrial oxidative-phosphorylation performance, whereas mitochondrial function is known to be reduced in HF^10–17,29,30^.

In addition, we found a significant reduction of cell stress markers GRP78, HSP90 and HSP70, as well as trend for fewer apoptotic cells in the hearts of cold-exposed pigs, when analyzed one weeks after MI. Furthermore, TNFα and TGFβ mRNA expression as well as iNOS and Arginase-1 protein abundance in tissue trended towards reduction, hinting at decreased local inflammation under hypothermic conditions, consistent with prior data^3^. This is supported by a significant reduction in iNOS detected by immunohistochemistry in the tissue of cold hearts. Moreover, we demonstrated that under cold conditions, lymphocytes extracted from splenic tissue showed significantly less proliferation in response to concanavalin-A, confirming that systemic inflammatory pathways are also attenuated after cold exposure^31^. As damaged mitochondria can release mitochondrial DNA (if not cleared efficiently by mitophagy) which acts as a ligand for TLR9 and the NLRP3 inflammasome, it is reasonable to suggest that autophagy/mitophagy triggered by hypothermia might limit inflammation. Decreased local and systemic inflammation would have a beneficial effect on remodeling, particularly fibrosis in the border zone, as we observed in the hearts of pigs treated with hypothermia, evident one week post MI.

In part, the beneficial effects may be attributed to reduced infarct size, as cold irrigation resulted in better reflow, and infarcts evolve over the first 24 hours^10^. However, there are several lines of evidence to suggest that autophagic flux is important to the improved outcome. First, hypothermia has a direct effect on autophagy as observed in mice subjected to MI and hypothermia (unpublished data). Secondly, we observed temperature-dependent differences in autophagy/mitophagy in the LV cores obtained from hearts at left ventricular assist device implantation. Thirdly, electron microscopic images obtained 3 hours post MI show differences in mitochondrial architecture and autophagy/mitophagy in the border zone. These early changes are unlikely to be explained by differences in infarct size or TIMI score. We were surprised that we did not see differences in autophagy/mitophagy in the remote zone despite the application of hypothermia, but this may indicate that autophagy/mitophagy are a response to tissue injury (therefore pertinent in border zone), and that the effect of hypothermia is to preserve the integrity of the autophagic machinery required for flux, rather than to initiate the process.

To investigate whether this response to hypothermia might also occur in human LV, we analyzed samples obtained during LVAD implantation, comparing samples from surgeries performed at different temperatures. Interestingly, we found a significant reduction of parkin, LC3-II and phospho-S6/S6 (Ser 235/236) ratio in whole lysate, consistent with our prior findings in the pigs and our hypothesis of a decrease in mTOR activity being a possible mechanism of action. Additionally, LC3-II and NDP52 in the mitochondrial fraction, followed a trend similar to that observed in our pig model. This suggests that during heart surgery under hypothermic conditions an increase in autophagy and mitophagy occurs, which we speculate might lead to repair and a chance for recovery. We performed multiple linear regression analysis for all patient characteristics. No patient characteristics besides core temperature significantly correlated with autophagy markers.

To conclude, our data implicate mitophagy and autophagy in the beneficial effects of TH on the myocardium. We showed translation to humans, where hypothermia is currently used in various clinical settings. Modulating the process of mitophagy or autophagy, could represent a new therapeutic approach to acute or chronic HF, the latter remaining a major cause of mortality^10,14^ and a persistent socioeconomic burden for health care^14^.

This study offers several translationally relevant findings. First, we show that administration of TH after the onset of reperfusion can mitigate post-MI remodeling. This increases its clinical utility, as it can be considered adjuvant therapy to be initiated sometime after the critical restoration of blood flow. Secondly, our study shows that autophagy/mitophagy represent a therapeutic target not only for acute MI^11–14^, but also for post-MI remodeling. It is likely that there exist many approaches to upregulate autophagy/mitophagy in addition to hypothermia, although it is yet unknown whether hypothermia confers additional benefits. Thirdly, this study shows that moderate hypothermia can trigger autophagy/mitophagy in both swine and humans. TH remains an important tool to further investigate the pathways involved in its beneficial effects on the myocardium. Furthermore, new therapies influencing mitochondrial turnover and autophagy might carry substantial benefit for increased recovery after acute and potentially reversible HF such as fulminant viral myocarditis, peripartum cardiomyopathy or MI with non-obstructive coronary arteries (MINOCA).

## Limitations of the Study

We are aware that the small sample size of most of our experiments (n = 3 per group) is a limitation of this study and our results should be interpreted with this limitation in mind. It is reassuring that results were internally consistent, lending confidence to our conclusion, namely the beneficial effect of hypothermia on myocardial tissue after infarction.

## Supplementary information

supplementary data

## Data Availability

All data acquired in this study as well as experimental details are available by contacting the corresponding author.
