# Supplementary material for: Myocardial hypothermia increases autophagic flux, mitochondrial mass and myocardial function after ischemia-reperfusion injury
Source: Sci Rep. 2019 Jul 10;9:10001. doi: 10.1038/s41598-019-46452-w (PMC6620356; doi:10.1038/s41598-019-46452-w)

Myocardial hypothermia increases autophagic flux, mitochondrial mass and myocardial function after ischemia-reperfusion injury

Stefanie Marek-Iannucci MD[1], Amandine Thomas PhD[1], Jean Hou MD[2], Annunziata Crupi PhD[4], Jon Sin PhD[1], David J Taylor PhD[1], Lawrence S Czer MD[1], Fardad Esmailian MD[1,3], Robert M Mentzer Jr MD[1], Allen M Andres PhD[1], Roberta A Gottlieb MD[1].

[1] Smidt Heart Institute, Cedars-Sinai Medical Center, Los Angeles, California, USA

[2] Department of Pathology, Cedars-Sinai Medical Center, Los Angeles, California, USA.

[3] Division of Cardiac Surgery, Cedars-Sinai Medical Center, Los Angeles, California, USA.

[4] Board of Governors Regenerative Medicine Institute, Cedars-Sinai Medical Center, Los Angeles, California, USA.

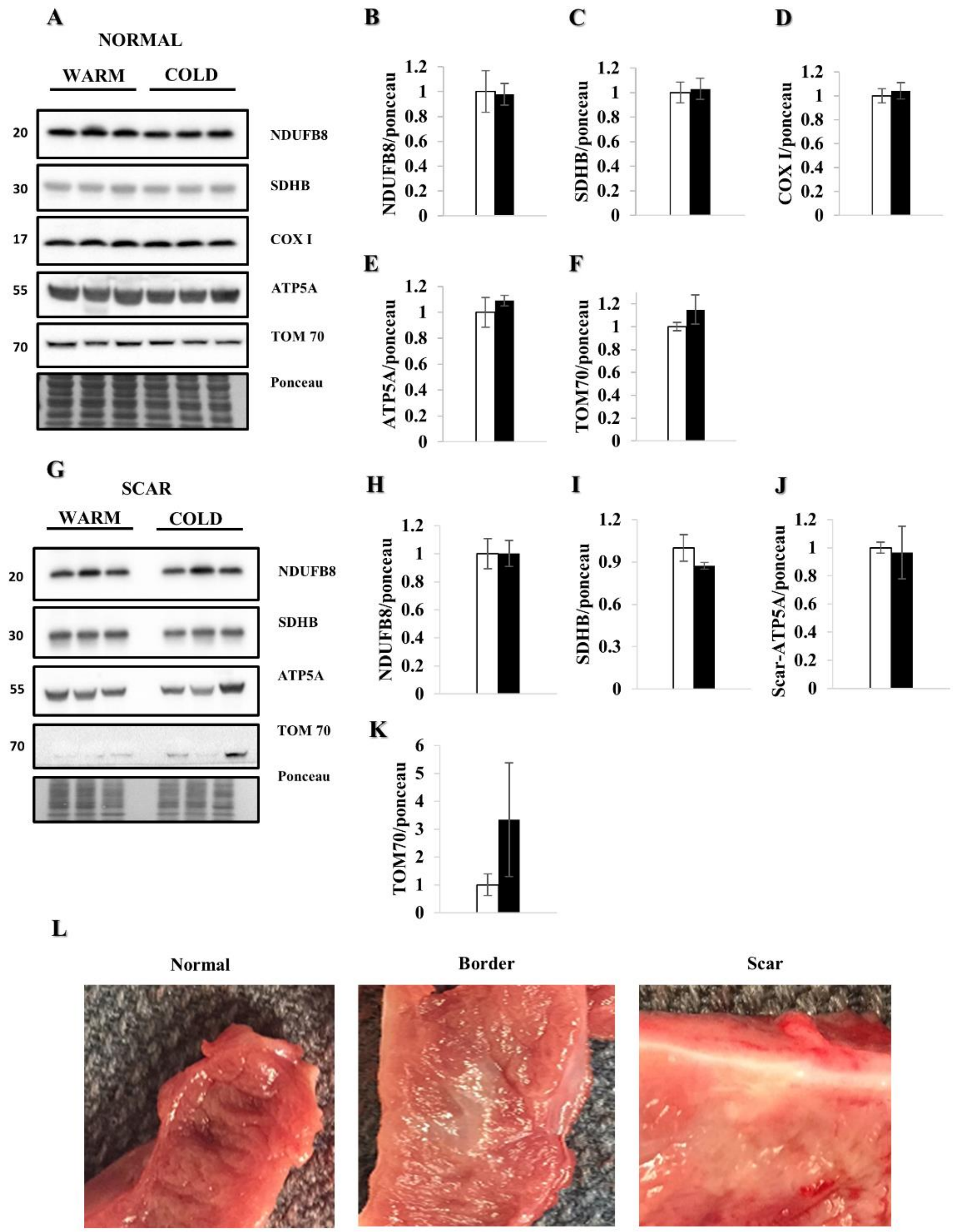

Supplemental Figure 1. Hypothermia in the setting of ischemia and reperfusion affects only the border zone. WB analysis were performed on tissue harvested one week post MI on female farm pigs (n=6). All proteins were normalized to ponceau. **(A)** WB analysis of the remote zone, with protein quantification **(B-F)** of NDUFB8, SDHB, COXI, ATP5A and TOM70 respectively. **(G)** WB analysis of scar tissue, with protein quantification **(H-K)** of NDUFB8, SDHB, ATP5A and TOM70 respectively. **(L)** Representative images of the freshly harvested tissue, demonstrating remote, border and scar zone respectively.

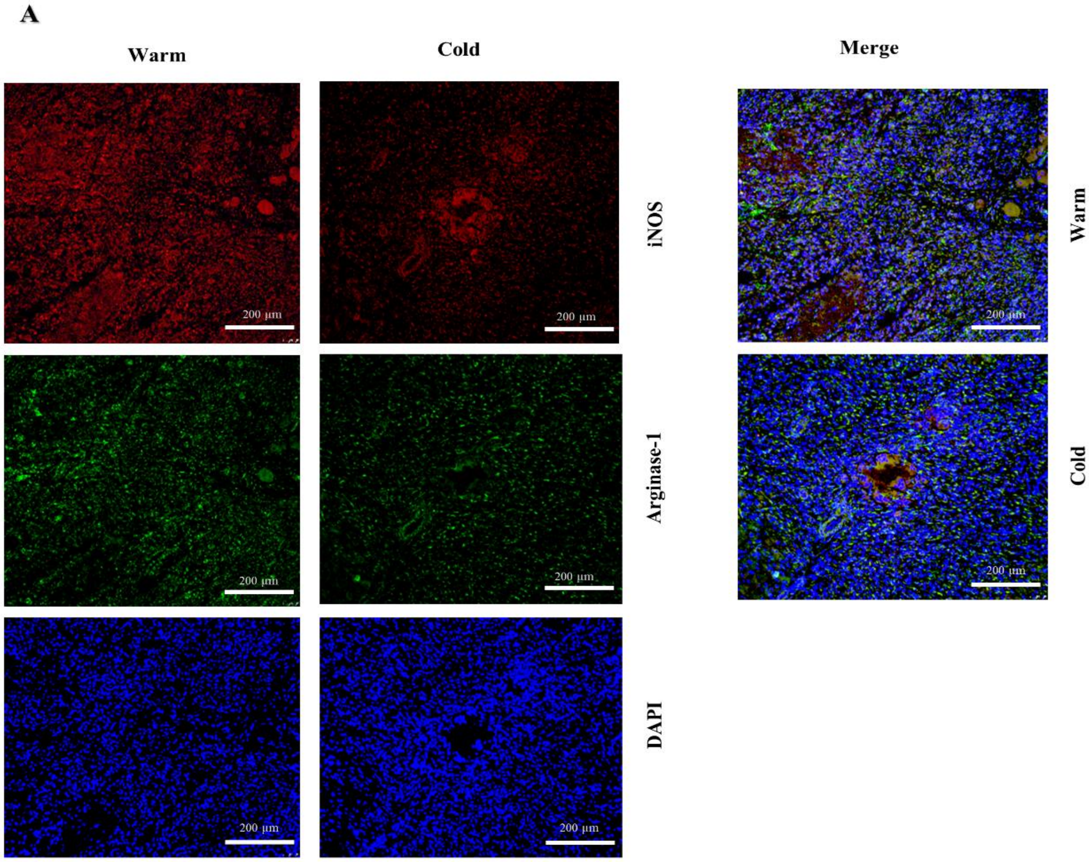

Supplemental Figure 2. Myocardial hypothermia reduces regional tissue inflammation. Myocardial border tissue of female farm pigs (n=6) was harvested one week post MI and further processed for histology. **(A)** Representative fluorescence microscopy images of iNOS, Arginase-1 and DAPI staining respectively.

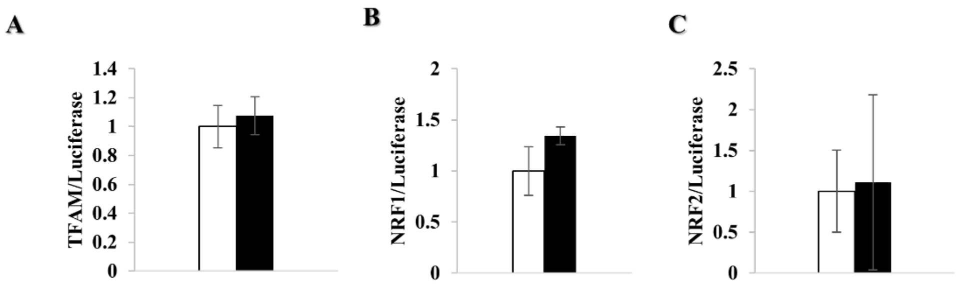

Supplemental Figure 3. Cold irrigation and its effects on mitochondrial transcription factors. To investigate whether the mitochondrial protein increase observed is related to simple accumulation or transcription we analyzed the expression of mitochondrial transcription factors TFAM, NRF 1 and NRF 2 with qPCR **(A-C)**. The data represents the mean ± SEM

Patient characteristics (n=13 total)	Warm group (n=6)	cold group (n=7)
Age (years ± SD)	59.2 ± 7.0	54.9 ± 9.6
Race		
Caucasian	n=4 (66%)	n=5 (71%)
African American	n=2 (33%)	n=2 (29%)
Sex (male)	n=6 (100%)	n=5 (71%)
Body Mass Index (kg/m2 ± SD)	34.7 ± 9	28 ± 3
Hyperlipidemia	n=4 (66%)	n=3 (43%)
Diabetes Mellitus Type 2	n=1 (17%)	n=1 (14%)
Obstructive Sleep Apnea Syndrome	n=3 (50%)	n=0 (0%)
Core temperature	36.4 ± 0.4	32.8 ± 0.5
Type of Cardiomyopathy		
ischemic	n=1 (17%)	n=1 (14%)
non-ischemic	n=5 (83%)	n=6 (86%)

Supplemental Figure 4. Baseline characteristic of patients included in this study. Table one shows the baseline characteristics of the patients included in this study. For age and Body Mass Index we calculated the mean age and its standard deviation (SD). For all other parameters a percentage within each group was calculated.

Full unedited gels

submission (SREP-18-49733A)

Full unedited gel for Figure 3

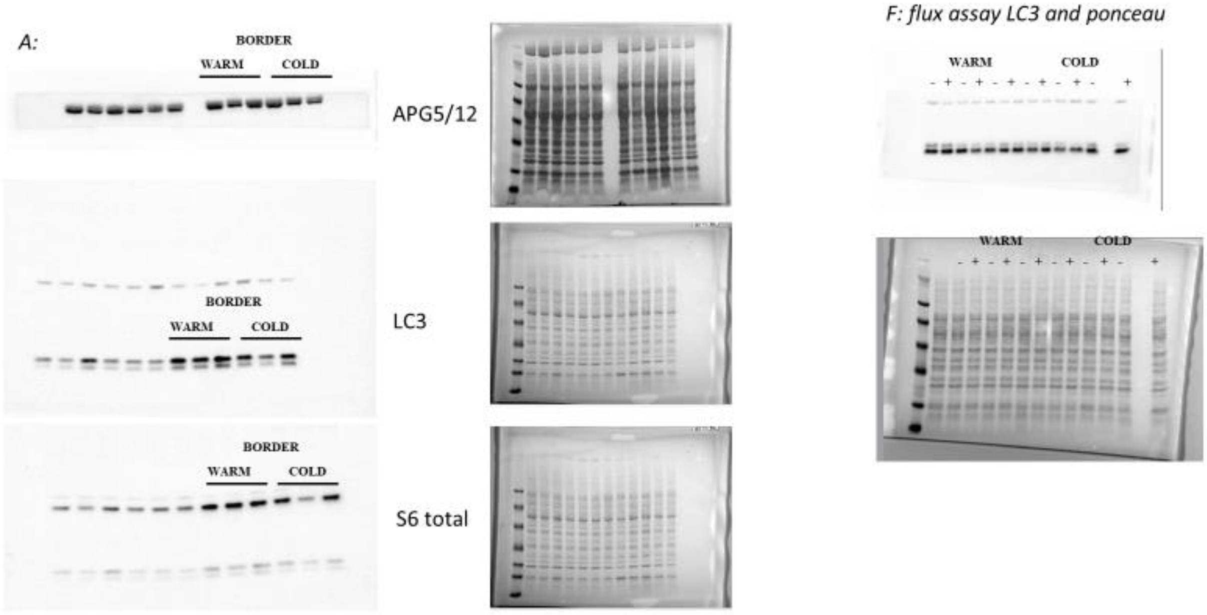

Full unedited gel for Figure 3 (continued):

A:

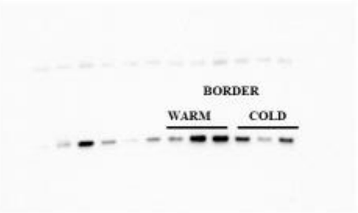

Phospho S6

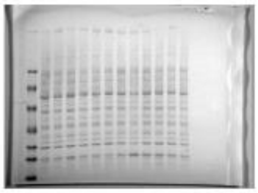

Full unedited gel for Figure 4

A:

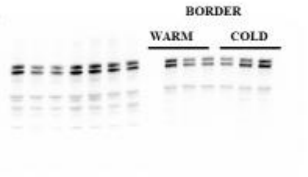

Opa-1

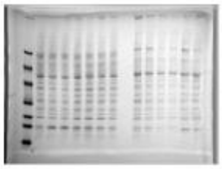

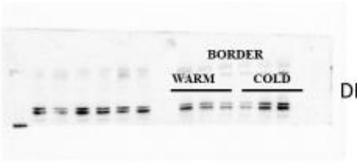

DRP-1

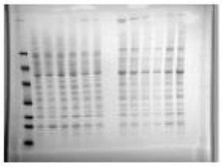

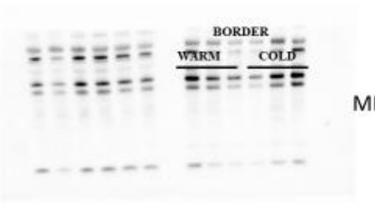

MFF

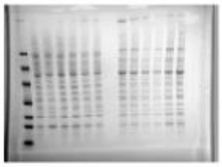

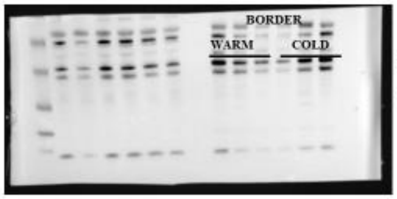

Merge MFF with ladder

Full unedited gel for Figure 4 (continued)

A:

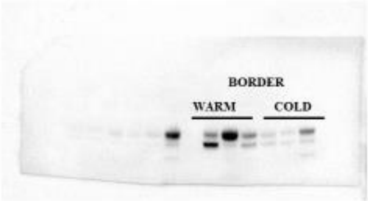

Mito-parkin

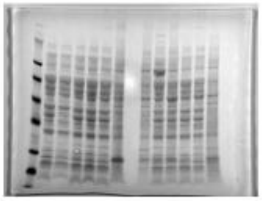

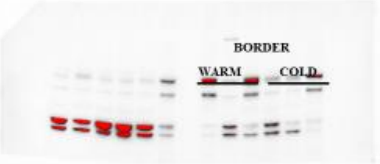

Mito-NDP52

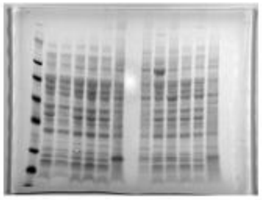

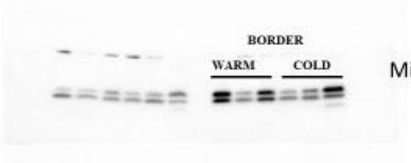

Mito-LC3

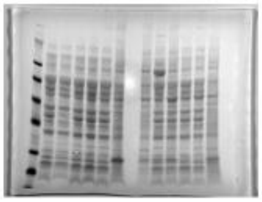

Full unedited gel for Figure 4 (continued)

A:

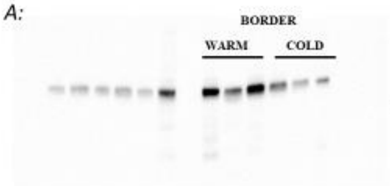

Mito-p62

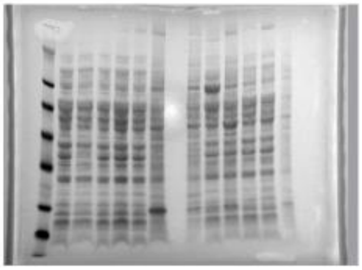

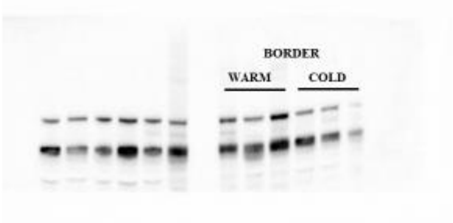

Mito-optineurin

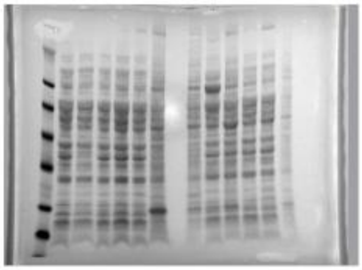

Full unedited gel for Figure 4 (continued):

A:

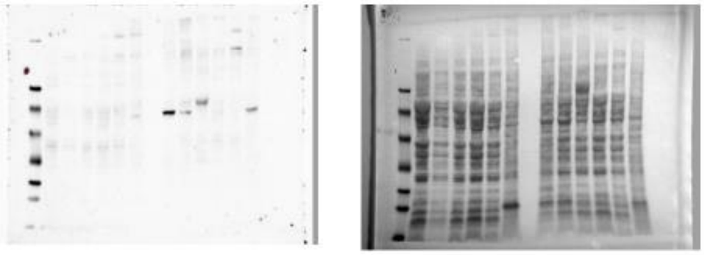

Mito - phospho-ubiquitin

Full unedited gel for Figure 5:

A:

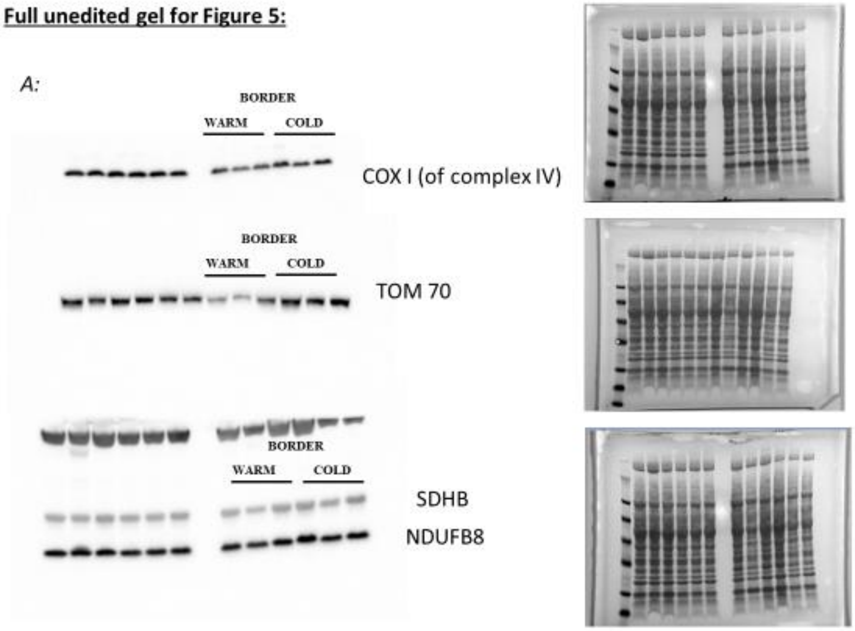

Full unedited gel for Figure 5 (continued):

A:

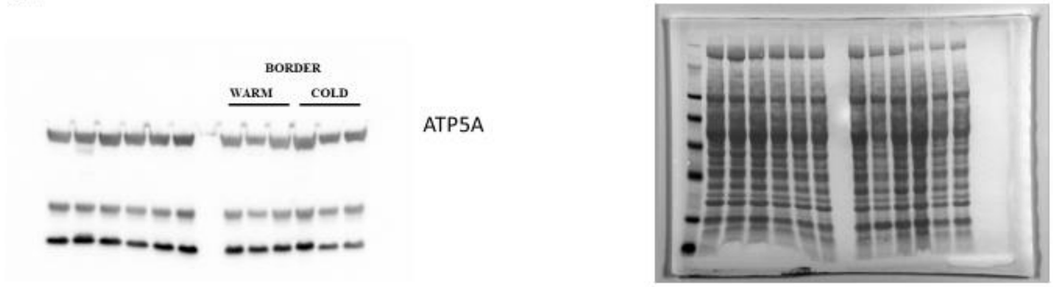

Full unedited gel for Figure 6

A:

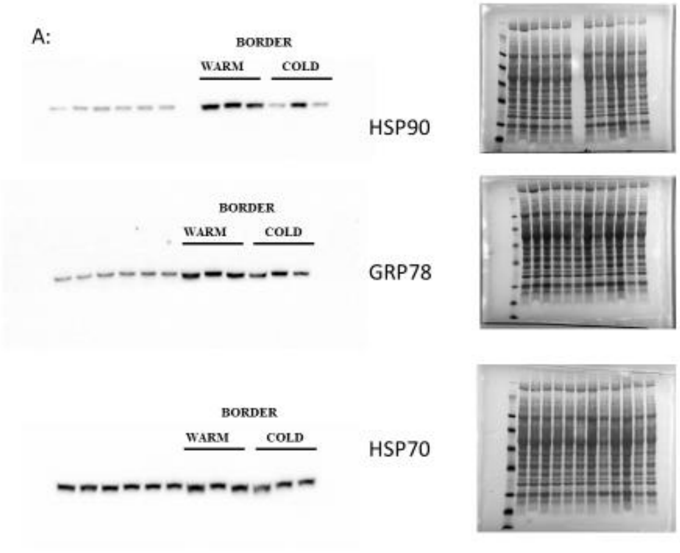

Full unedited gel for Figure 7:

D:

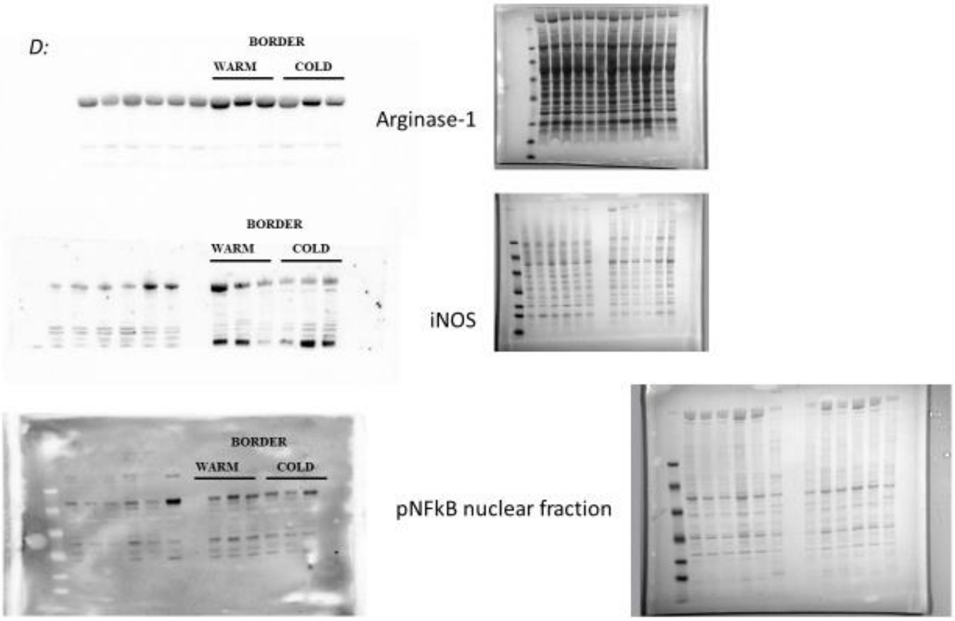

Full unedited gel for Figure 7 (continued):

D:

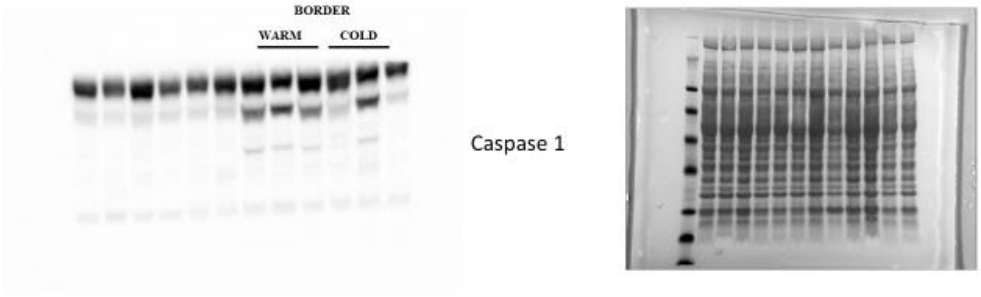

Full unedited gel for Figure 9:

A:

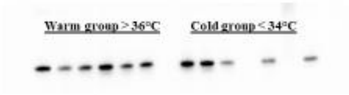

S6 total VAD samples

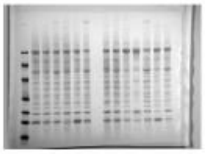

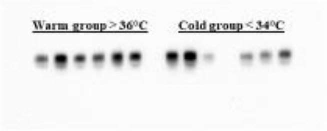

Parkin VAD samples

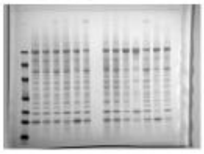

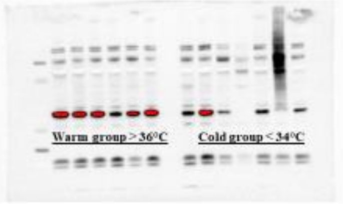

LC3 VAD samples

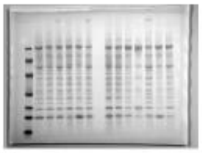

Full unedited gel for Figure 9 (continued):

A:

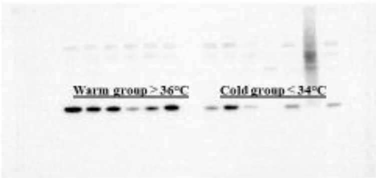

Phospho S6 VAD samples

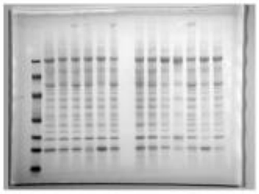

B:

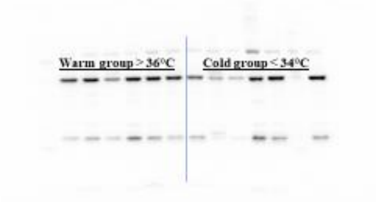

Mito-NDP52 VAD samples

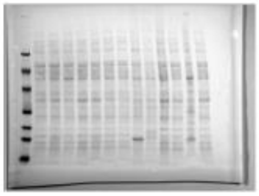

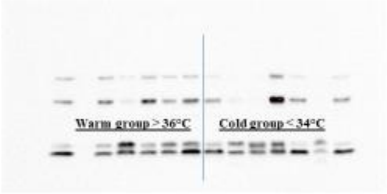

Mito-LC3 VAD samples

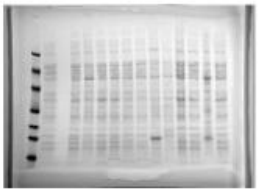

Supplement: Supplementary file 1 — supplementary data [file 41598_2019_46452_MOESM1_ESM.pdf]
